# Core needle biopsy-derived breast cancer microtumors enable functional drug-response testing for precision oncology

**DOI:** 10.1016/j.isci.2026.117914

**Published:** 2026-10-15

**Authors:** Mooktapa Plikomol, Suphawat Laohawiriyakamol, Piyanun Wangkulangkul, Paramee Thongsuksai, Pokphon Khirilak, Ratsamaporn Nontikarn, Onpawee Phanthuvet, Karan Ulhaka, Pasarat Khongkow

**Affiliations:** 1 Department of Biomedical Sciences and Biomedical Engineering, Faculty of Medicine, Prince of Songkla University, Songkhla 90110, Thailand; 2 Department of Surgery, Faculty of Medicine, Prince of Songkla University, Songkhla 90110, Thailand; 3 Department of Pathology, Faculty of Medicine, Prince of Songkla University, Songkhla 90110, Thailand; 4 Translational Medicine Research Center, Faculty of Medicine, Prince of Songkla University, Songkhla 90110, Thailand

**Keywords:** breast cancer, patient-derived microtumors, PDMs, cancer precision medicine, drug response prediction

## Abstract

To predict neoadjuvant chemotherapeutic outcomes, breast cancer organoids have been developed. However, low yields from small tissue specimens and the high cost of niche factor-enriched medium remain barriers to clinical translation. Here, breast cancer patient-derived microtumors (PDMs) from core needle biopsies and small tissue specimens were established using a modified culture protocol and an optimized fetal bovine serum (FBS)-based culture medium, achieving a 75% success rate (6 of 8 cases). PDM characteristics mostly agreed with the tissue origins, according to growth evaluation, histopathology, immunohistochemistry, and genetic profiling. As a proof of concept, PDMs were tested with neoadjuvant chemotherapy alongside the patients’ treatment. Notably, the PDM results were positively correlated with the patient outcomes and were obtained within a clinically relevant time frame (∼2–3 weeks). Collectively, our protocol and FBS-based medium efficiently generate breast cancer PDMs from core needle biopsies and small tissue specimens, with promising potential to guide neoadjuvant chemotherapy decisions.

## Highlights

• Microtumors from core needle biopsies and small specimens achieved a 75% success rate
• A serum-based medium with fewer niche factors supported short-term microtumor culture
• Microtumors mostly recapitulated the histological and genetic features of original tumors
• Microtumor drug responses potentially predicted patient outcomes within two to three weeks

## Graphical abstract

**Figure:**
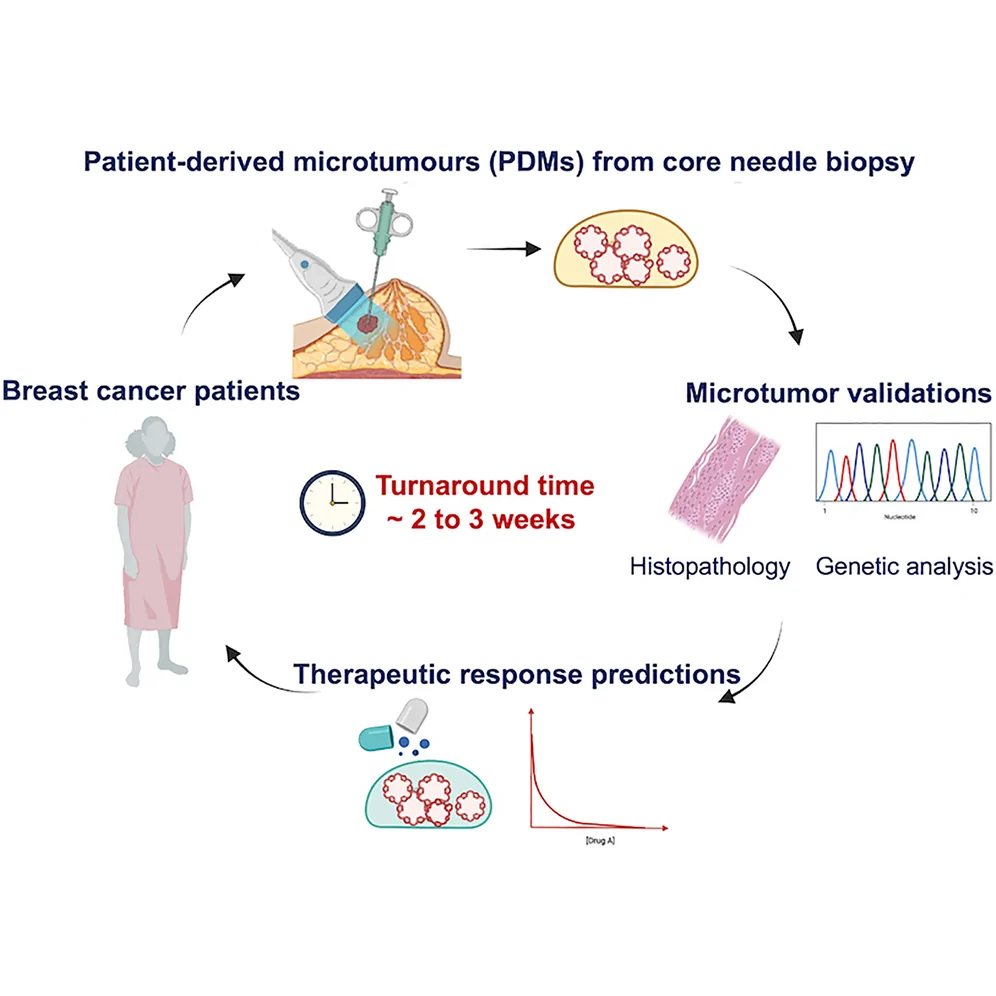

## Teaser

Cell biology; Cancer

## Introduction

Breast cancer is the most commonly diagnosed cancer among females worldwide, accounting for approximately 24% of all emerging cancer cases.^1^ It is a heterogeneous disease that comprises many subtypes, based on genetic profiling, histopathology, and the clinical outcomes of individual patients.^2^ The various nature of breast cancer initiation means that tumor development and progression do not follow a fixed course, and tumor mass is composed of several cell subclones with different drug sensitivities. Unsurprisingly, almost 60% of patients with breast cancer suffer from cancer progression or recurrence.^3^ Thus, the disease should rather be clarified as the integrated analysis of key cellular processes and applied to personalized therapy.^4^

To overcome the clinical challenge of cancer heterogeneity, precision medicine is an approach that provides optimized medical treatments to individual patients, based on their genetic alterations, histopathological characteristics, and appropriate stratification.^5^ The identification of driver mutations and predictive biomarkers paves the way for targeted therapies. For example, patients with breast cancerwith the estrogen receptor-positive subtype and the human epidermal growth factor receptor 2 (HER2)-positive subtype are mostly treated with hormone therapies and anti-HER2 blockade agents, respectively. Nevertheless, not all patients with these cancer subtypes respond well to the biomarker-specific therapies.^6^ To better elucidate cancer heterogeneity, next-generation sequencing is an important technique to perform comprehensive genomic profiling in individual patients to guide potential targeted therapies, such as Olaparib for breast cancer with *BRCA1/2* mutations.^7^ Although the sequencing technique is promising, tumors with clinically actionable mutations still exhibit varying responses to targeted therapies. This is possibly associated with a lack of complete information regarding differential cellular states and genetic backgrounds. Furthermore, the substantial proportion of tumors does not possess any known clinically actionable alterations.^8^^,^^9^ Therefore, drug response prediction models have been developed to be utilized for mimicking patient’s tumor and predicting clinical outcomes.

Over the last decade, several types of personalized cancer models have emerged, which primarily aim to effectively capture breast cancer characteristics and improve the accuracy of therapeutic prediction.^10^^,^^11^ Conventionally, a classic monolayer culture model provides cost-effective, less time-consuming, and simplified methods for performing drug screening. However, the model is unable to recapitulate the complex biochemical- and biophysical factors of *in vivo* tumor microenvironment.^12^ Next, a patient-derived xenograft (PDX) model is generated by transplanting breast cancer cells, derived from patients, into immunodeficient mice. The xenograft model capably mimics patients’ tumor profiles and drug responses,^13^ but it still has critical drawbacks that obstruct clinical translation. These include high experimental cost, long processing time, low-throughput drug testing, and ethical concerns.^10^^,^^14^

Inspired by three-dimensional cell cultures, breast cancer patient-derived organoids (PDOs) have been developed to bridge the gaps of previous models. The organoids are established from the tissue specimens of individual patients, which contain cancer stem cells and the native tumor microenvironment.^13^^,^^15^^,^^16^^,^^17^^,^^18^ The important key to generating breast cancer organoids is a niche factor enriched-culture medium such as Neuregulin 1, R-spondin, FGF7/10, Noggin, Y-27632, and A83-01.^13^ Because of the high cost of the niche cocktail, the next-generation organoid concept aims to minimize and simplify the recipes of organoid culture medium both fetal bovine serum (FBS)-based medium and FBS-free based medium.^19^^,^^20^^,^^21^^,^^22^ In addition, to be feasible in clinical settings, tissue specimen requirements for organoid generation need to be minimal. In particular, a core needle biopsy is the only definitive way to diagnose breast cancer following the breast imaging-reporting and data system (BI-RADS) and to guide the medical team in creating a neoadjuvant treatment plan.^7^^,^^23^^,^^24^ However, the efficiency of generating organoid from such limited specimens remains challenging, due to the preferential outgrowth of normal epithelial cells during culture.^16^^,^^18^^,^^25^^,^^26^

Here, we established breast cancer patient-derived microtumors (PDMs) from core needle biopsies and small tissue specimens using a modified culture protocol and an optimized FBS-based medium. The PDMs recapitulated both the original tumor characteristics and the patients’ responses to neoadjuvant chemotherapy, supporting their potential as predictive models to guide treatment decisions. Unlike conventional organoid platforms, which rely on dissociated cells that self-organize and expand through long-term passaging,^13^^,^^17^^,^^18^ our PDMs were derived from intact or non-fully dissociated tumor fragments, preserving native tumor architecture while shortening the generation time. Accordingly, we designate them as ‘microtumour or *ex vivo* tumor fragment models,’^27^^,^^28^^,^^29^ which are suitable for a short drug screening turnaround time.

## Results

### Improving culture protocol for developing breast cancer patient-derived microtumors (PDMs) from core needle biopsies and small tissue specimens

Breast cancer tissues were obtained from 6 treatment-naive patients and 1 relapse patient in a total of 8 cases. The specimens were derived from variable receptor statuses, including 4 cases of tumor positive for estrogen receptor (ER) and/or progesterone receptor (PR), 3 cases of tumor positive for human epidermal growth factor receptor (HER2), and 1 case of triple-negative breast cancer. They were divided based on histological subtypes into 6 cases of infiltrating ductal carcinoma (IDC) and 2 cases of mixed mucinous infiltrating ductal carcinoma (MC-IDC) among 2 to 3-grading scores. The specimens were sourced from the 6 cases of core needle biopsy and the 2 cases of mastectomy (Tables 1, S1, and S2). The success criteria for PDM derivation consisted of more than or equal to 50 isolated tumor fragments from mechanical or enzymatic digestion, and at least 30 active PDMs, capable of tracking growth during a 1–2-week expansion period, were observed. Regarding these criteria, 6 of 8 cases, accounting for 75% of all specimens, were successful for PDM generation. Categorized by surgical type, all cases for mastectomy-derived specimens and 4 of 6 cases for biopsy-derived specimens achieved PDM derivation, with 100% and 67% success rates, respectively. Subsequently, the 4 successful cases were utilized for histological and genetic validations and testing of neoadjuvant chemotherapy, compared with the matched patients.

**Table 1:** Core needle biopsies and small tissue specimens enrolled in this study (*N* = 8 specimen cases, derived from 7 patients; see more details of tissue characteristics and patients’ medical reports in Tables S1 and S2)

Specimen cases	Surgical types	Patient ages	Clinical statuses	AJCC staging	Histological diagnoses	Histological grades	ER/PR/HER2-based classifications
BRC16	biopsy	49	new case	4	IDC	2	hormone receptor-positive subtype (ER+/PR+/HER2-)
BRC17	biopsy	71	relapsed case	2B	IDC with mucinous	3	hormone receptor-positive subtype (ER+/PR-/HER2-)
BRC18	biopsy	52	new case	4	IDC	2	hormone receptor-positive subtype (ER+/PR+/HER2-)
BRC19	mrm	84	new case	3B	IDC	2	triple-negative subtype (ER-/PR-/HER2-)
BRC20	biopsy	56	new case	3B	IDC	3	HER2-positive subtype (ER-/PR-/HER2+)
BRC21	biopsy	48	new case	3B	IDC	2	HER2-positive subtype (ER + but low/PR-/HER2+)
BRC22	biopsy	54	new case	2B	IDC	3	HER2-positive subtype (ER+/PR+/HER2+)
BRC17-2	mastectomy	72	relapsed case	2B	IDC with mucinous	3	hormone receptor-positive subtype (ER+/PR-/HER2-)

Note: MRM = modified radical mastectomy, IDC = invasive ductal carcinoma, AJCC = American Joint Committee on Cancer, ER = estrogen receptor, PR = progesterone receptor, HER2 = human epidermal growth factor receptor.

To enhance the achievement of PDM establishment, a simple technique and two optimal steps were developed in our culture protocol following a workflow in Figure 1A. Initially, core needle biopsies and small tissue specimens were minced in a cold working medium. During this process, the chance of microtumor derivation could be estimated under a light microscope by the observation of releasing fragments and cell clusters in the medium. According to our observation, the tumor fragments and cell clusters were found in the medium during the mincing step in about 5 of 6 successful cases of PDM generation (BRC16, 17, 19, 21, and 17–2 cases). In contrast, the failure cases (BRC18 and 22 cases) had neither cell clusters nor tumor fragments released into the medium. To clarify this phenomenon, the tissues were stained with hematoxylin and eosin (H and E) to depict their histological features. In correlation with our observation, the tissues that were successful for PDM derivation had many epithelial cells with distinct nuclei and cytoplasm, but the failure cases had few cells with unclear morphology (Figure 1B).

**Figure 1:**
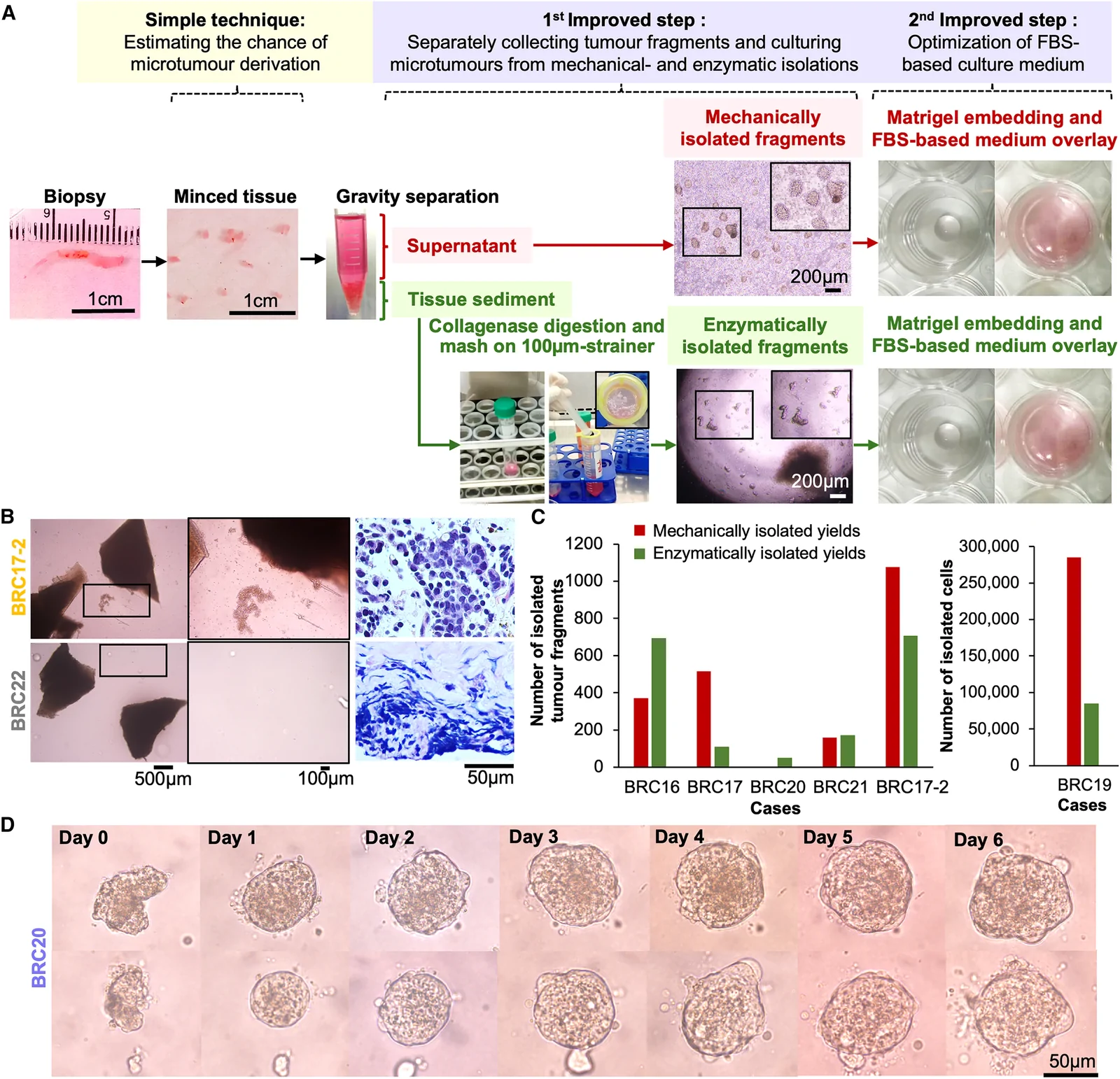
Improving culture protocol for developing breast cancer patient-derived microtumors (PDMs) from core needle biopsies and small tissue specimens (A) The workflow of the PDM culture protocol presents the overall process, starting from tissue specimen enrollment, mincing, the chance estimation of microtumor derivation by observing fragment and cell cluster release, gravity separation, the collection of mechanically isolated fragments from the supernatant, the enzymatic digestion of tissue sediment, the collection of enzymatically isolated fragments, and culturing in FBS-based medium. (B) The representative color bright-field images displayed tumor fragments and cell clusters that were released into a working medium during the mincing process. To visualize tissue characterization, Hematoxylin and Eosin (H and E) staining was performed and compared between the successful and failed cases of microtumor derivation (*N* = 6 analyzed cases for histological analysis; scale bars, 500 μm (left), 100 μm (middle), and 50 μm (right). (C) The bar charts present the number of tumor fragments from all successful cases of PDM establishment and the number of cells from BRC19 cases, compared between mechanical and enzymatic isolations. (D) The representative color bright field images of PDM growth from day 0 to day 6 of culture (scale bars, 50 μm).

Firstly, to enhance the achievement of PDM establishment, mechanically isolated and enzymatically isolated fragments were separately collected. This step was different from the conventional organoid culture protocols of previous studies,^13^^,^^15^^,^^17^^,^^18^^,^^30^^,^^31^ which only harvested tumoroid fragments from enzymatic digestion. Regarding our results in Figure 1C, the yields of tumor fragments were variable between mechanical- and enzymatic digestions, depending on tissue specimens. In addition, although tumor fragments were primarily required, the BRC19 tissue specimen gave the majority of single cells, which was possible due to the tissue properties of the triple-negative subtype, such as weak cell-cell junctions and loss of cell adherence.^32^ By comparison, mechanical isolation resulted in tumor fragments or cell density higher than another method in about 3 cases (BRC17, BRC19, and BRC17-2 cases), while enzymatic isolation was higher in 2 cases (BRC16 and BRC21 cases). Also, BRC20-tumor fragments were derived from enzymatic isolation only. Because of this variability, to avoid yield loss, tumor fragments or cell suspensions from mechanical and enzymatic isolation were subsequently cultured and maintained separately.

Next, to generate breast cancer PDMs, fetal bovine serum (FBS)-based culture medium was optimized and utilized to support their growth, the expression of morphological phenotypes, and survivability. A key principle was the inhibition of the Rho-associated protein kinase (ROCK) pathway, which was associated with promoting apoptosis. Underlining this principle, the ROCK inhibitor, also known as Y-27632, was added to our medium as an essential component for stimulating tumoroid proliferation and preventing cell apoptosis.^13^^,^^22^^,^^33^^,^^34^^,^^35^ Referring to our results, 6-day-cultured PDMs under Y-27632-supplemented medium had a significant increase in diameter sizes with a 12.4-fold growth rate based on area, compared to the control (Figures 2A and 2B). In addition, while the supplemented medium induced the expression of cystic microtumors about 37% of all analyzed PDMs, the control exhibited a phenotype dropping to only 13% (Figures 2C and 2D). For maintaining microtumor viability, the supplemented medium demonstrated that the PDMs still contained a high density of living cells and few dead cells despite over 2 to 3 weeks of culture, confirmed by Calcein AM (live indicator; green) and BOBO-3 iodide (dead indicator; red) staining (Figure 2E).

**Figure 2:**
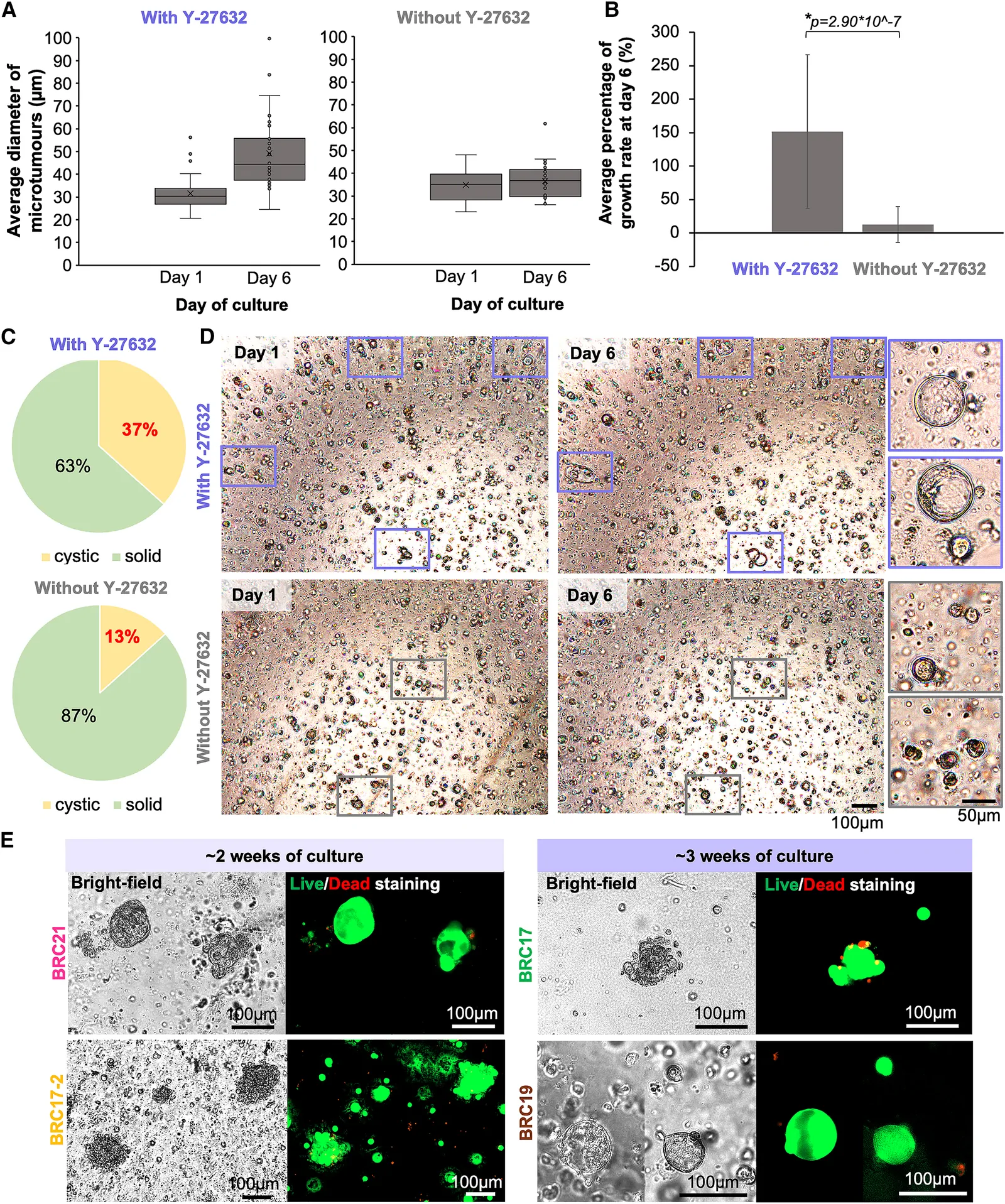
Optimization of FBS-based breast cancer PDM culture medium by adding the Rho-kinase inhibitor Y-27632 (A) The box and whisker plots and (B) the bar chart show that Y-27632-supplemented medium resulted in a higher increase in both PDM size and growth rate after 6 days of culture, compared to the control (mean ± SD from *N* = 30 analyzed microtumors of BRC19 case; statistical analysis by Welch’s unpaired *t* test; *p* value = 2.90 × 10^−7^). (C) The pie charts and (D) the representative color-brightfield images demonstrated that Y-27632-supplemented medium better induced PDMs to express cystic formations than the control (*N* = 30 analyzed microtumors of BRC19 case; values are the percentages of microtumors with cystic formation; scale bars, 100 μm and 50 μm for the low- and high-magnification images, respectively). (E) The representative images of calcein AM (live indicator in green color) and BOBO-3 iodide (dead indicator in red color) staining confirmed living PDMs, containing a high density of viable cells, although they were cultured in Y-27632-supplemented medium over 2 to 3 weeks after isolation (*N* = 4 analyzed cases; scale bars, 100 μm).

### Breast cancer PDM derivations and growth analyses

To investigate growth capacity, individual PDMs were monitored daily after isolation to track their formation duration, morphological phenotypes, and growth rates. After isolation, tumor fragments looked like irregular in shape and could form a spherical shape within a day. Then, the fragments continuously increased in size and exhibited the signs of progression, such as protrusion, homogeneous opacity, well-defined edges, and three-dimensional structure (Figure 1D). The variable phenotypes of PDM morphologies could be observed within 6 days of culture, and the generated PDMs from mechanical and enzymatic digestions were similar in their expression. There were mixed phenotypes (solid, cystic, and/or polycystic patterns) in BRC19 and BRC21 cases and the solid/dense phenotypes in the BRC17-2 case (Figure 3A). For growth quantification, the PDMs from 6 successful cases had growth rates based on area in the range of 16.9–172.6% after 6-day culturing, depending on the characteristics of tissue origins (Figure 3B). Notably, BRC17 and BRC17-2 PDMs, derived from the same patient relapse with different times and surgical types of tissue specimens, slowly grew during expansion periods. This was correlated with the patient who was diagnosed by clinicians with slow-growing malignancy, based on 7 year-ultrasound tracking (Figure 6D). In addition, when comparing mechanically- and enzymatically isolated PDMs, they had no significant difference in growth rates and had a resemblance in increasing size ranges and growth patterns (Figures 3C and 3D).

**Figure 3:**
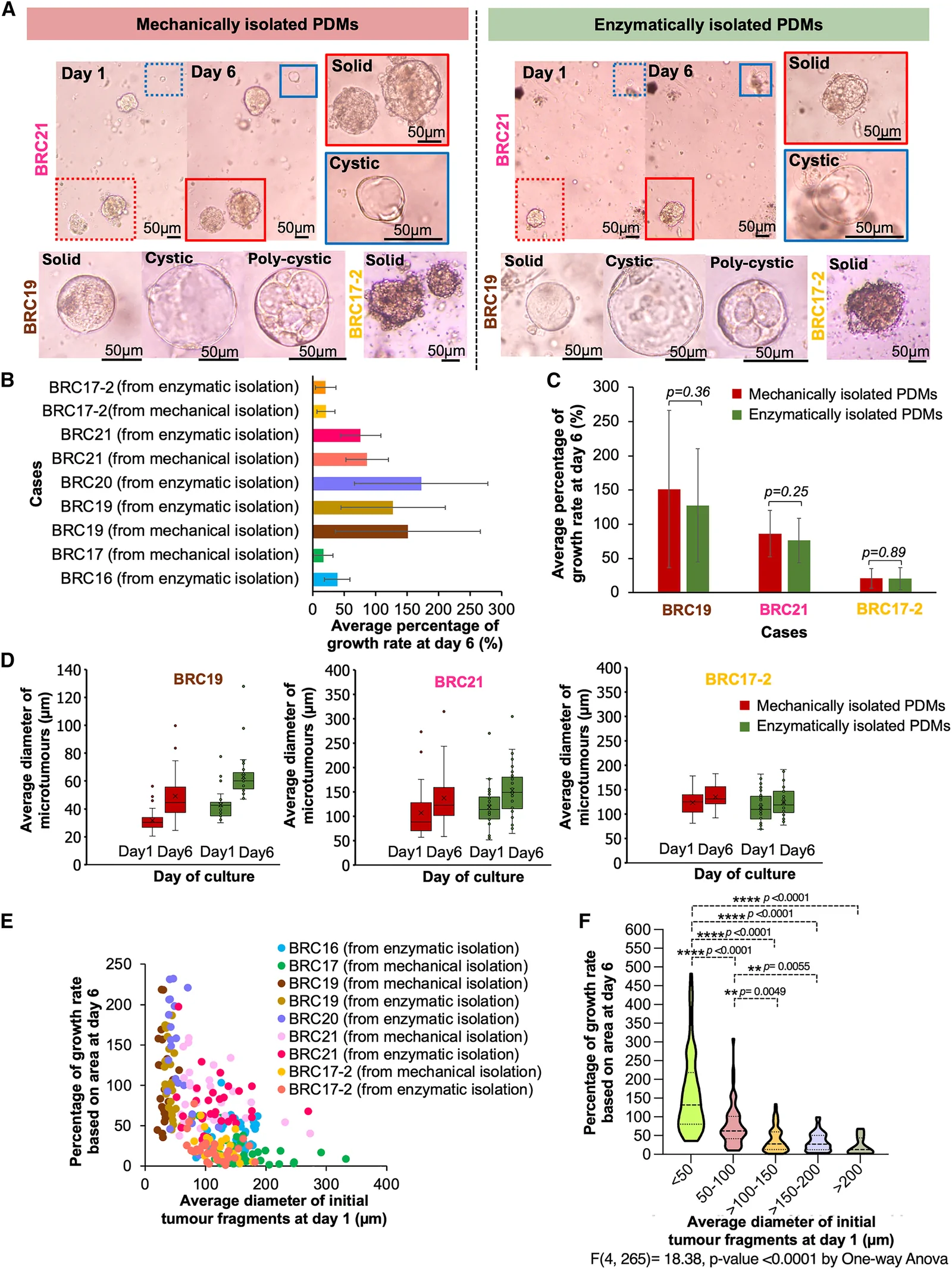
Breast cancer PDM derivations and growth assessments (A) The representative color bright-field images of mechanically and enzymatically isolated PDMs displayed the similarity of morphological expressions (*N* = 3 analyzed cases; scale bars, 50 μm). (B) The bar chart showed the growth rates based on area of mechanically- and enzymatically isolated PDMs after 6-day culturing in all cases (mean ± SD from *N* = 30 analyzed microtumors in each case). (C) The bar chart presented the PDM growth rates of both isolation methods, which showed no significant difference each other (mean ± SD from *N* = 30 measured microtumors in each case and statistical analysis by Welch’s unpaired *t* test; *p*-value = 0.36, 0.25, and 0.89 for BRC19, BRC21, and BRC17-2, respectively). (D) The box and whisker plots showed the similar size ranges and growth behaviors between mechanically and enzymatically isolated PDMs after 6 days of culture (mean ± SD from *N* = 30 analyzed microtumors in each case). (E) The dot plot and (F) the violin plot illustrated the influence of initial fragment sizes on PDM growth rates after 6-day culturing. Statistical analysis was performed using ordinary one-way Anova followed by Tukey’s multiple comparisons test for indicating significant level between groups (*N* = 270 analyzed microtumors in total from 6 successful cases, including *N*_<50μm_ = 74, *N*_50–100μm_ = 57, *N*_>100–150μm_ = 84, *N*_>150–200μm_ = 45, and *N*_>200μm_ = 10; F(4,265) = 18.38, *p*-value < 0.0001).

To shorten the interval to the drug assay, a fragment-based culture was primarily performed in this study. Following tracking PDM growth, we observed that the diameter sizes of initial fragments could affect the subsequent growth rates. After a 6 days-culturing, the average growth rates of PDMs, started with fragment sizes of less than 50 μm, 50 to 100 μm, more than 100 to 150 μm, more than 150 to 200 μm, and more than 200 μm were 162%, 77.34%, 38.75%, 32.96%, and 21.94%, respectively. When focusing on the group of less than 50 μm and the group of 50–100 μm, these initial tumor fragment sizes exhibited statistically significant differences for the following growth rates, compared to others. Therefore, the majority of initial fragments in this study were limited to size ranges of less than or equal to 100 μm in both mechanical and enzymatic isolations to ensure their growth capacity (Figures 3E and 3F).

Regarding our modified culture protocol, breast cancer PDMs could be generated from core needle biopsies and small tissue specimens, as small as a single biopsy (BRC20 and BRC21 cases) or with a minimum volume of ∼0.1 × 0.1 × 0.7 cm^3^ (BRC17 case). The generated PDMs were capable of expansion which respected the behavior of tumor origin and demonstrated specific morphological phenotypes in individual cases (Figure 4).

**Figure 4:**
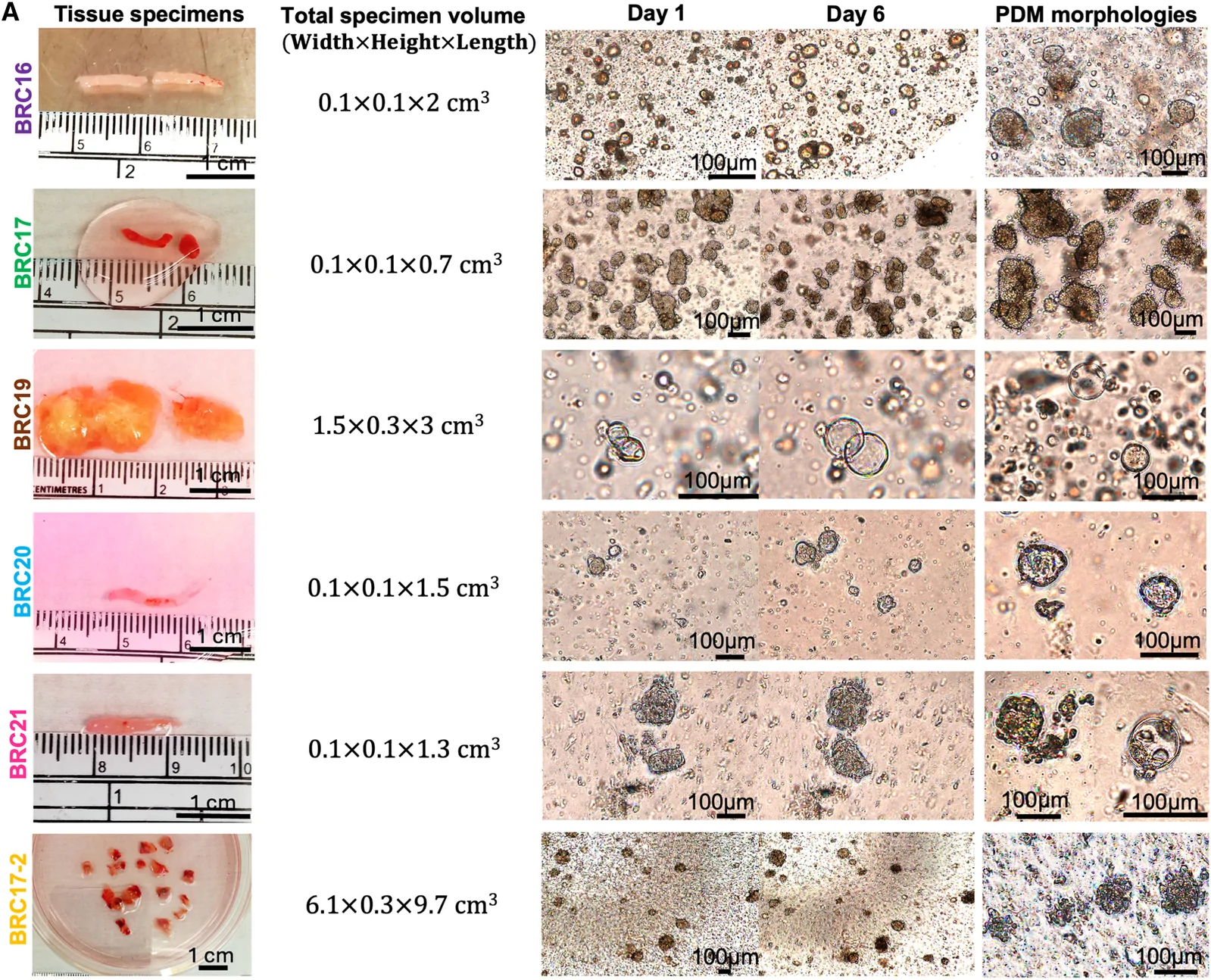
Feasibility of breast cancer PDM culture, performed with the variable tissue volumes of core needle biopsies and small tissue specimens, using a modified culture protocol Breast cancer specimens that were subsequently capable of PDM establishment alongside their respective total volumes (scale bars, 1 cm). In addition, the generated PDMs in each case exhibited distinct microtumor growths and morphological phenotypes, as demonstrated by the color brightfield images of microtumors on days 1 and 6 of culture (scale bars, 100 μm).

### Preserving the original tissue characteristics of breast cancer PDMs

To prove that the generated PDMs could recapitulate the tumor heterogeneity of tissue origins, histological- and genetic profiling was performed. Following histological evaluation with hematoxylin and eosin (H and E) staining, the original tissues presented with tumor epithelial cells surrounded by mesenchymal connective tissue. In contrast, the generated PDMs, derived from mechanical- and enzymatic isolations, exclusively showed the organization of tumor epithelial cells. Also, the PDMs were observed some typical cancerous features,^13^^,^^36^ including cohesive microtumors with enlarged and polymorphic nuclei (BRC17, 21, and 17–2 cases) and discohesive microtumors with vacuole formations (or signet ring-like cells), mitotic figures, and high pleomorphism (BRC19 case). Furthermore, as demonstrated by immunohistochemical detection, the generated PDMs, derived from both isolations, could recapitulate together estrogen receptor (ER), progesterone receptor (PR), and human epidermal growth factor receptor 2 (HER2) statuses consistent with the matched tissues. For instance, the mechanically- and enzymatically isolated microtumors of BRC21 and BRC17-2 cases exhibited strong positivity for HER2 and ER expressions, respectively, corresponding to their tissue origins (Figure 5A).

**Figure 5:**
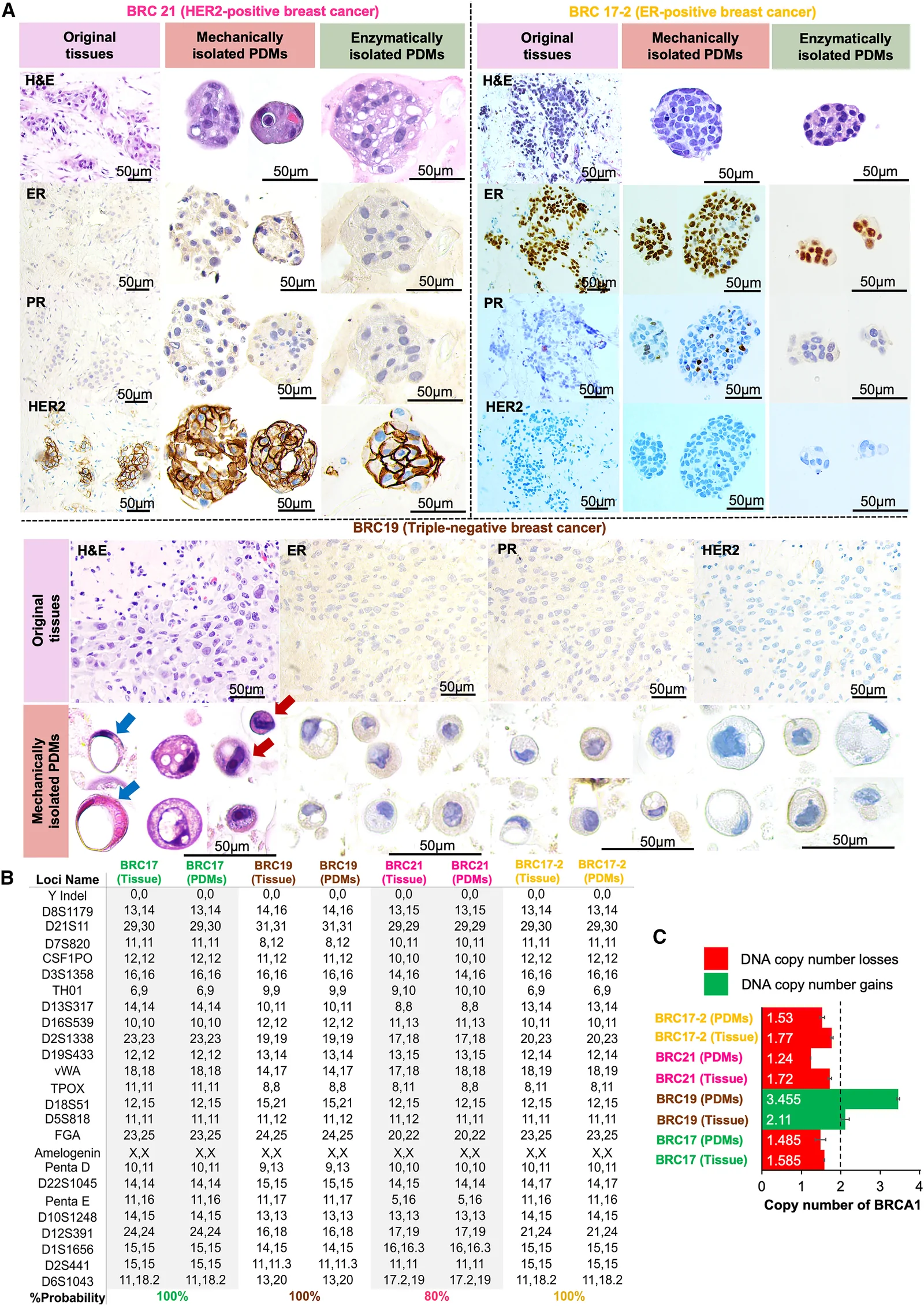
Histological and genetic characterizations of breast cancer PDMs, compared to the tissue origins (A) The representative results of histological and immunohistochemical analyses were compared among tissue origins, mechanically and enzymatically isolated PDMs (*N* = 4 analyzed cases for histological analysis and *N* = 3 analyzed cases for immunohistochemical analysis, ER: Estrogen receptor, PR: progesterone receptor, and HER2: human epidermal growth factor receptor). The blue and red arrows depicted signet ring-like cells and mitotic figures, respectively, which were associated with the histological features of tumor cells in the aggressive stage and poor prognosis. All results were presented by the montage images of multiple microscopic fields to illustrate the heterogeneity of microtumor phenotypes within the same culture batches (scale bars, 50 μm). (B) The results of short tandem repeat (STR) profiling showed the identification of tissue-microtumor pairs among 25 identified loci on different chromosomes (*N* = 4 analyzed cases). (C) The bar chart demonstrated the copy number alterations (CNAs) of PDMs, correlated to the original tissues (the negative cut-off of CNAs is 2: mean ± SD from duplicates of 4 analyzed cases).

In another, to authenticate the individual patient origins of generated PDMs and exclude cross-contamination or sample mix-up, short tandem repeat (STR) analysis was performed. Based on the results, the microtumor and tissue-pairs of BRC17, BRC19, and BRC17-2 cases were similar to each other in 25-locus STR profiling, demonstrating 100% probability. However, the BRC21 case showed approximately 80% probability with mostly single-step mutations, presumably arising from tumor mosaicism and genetic instability^37^^,^^38^ (Figure 5B). Moreover, the microtumor and tissue pairs were investigated copy number alterations (CNAs) of *BRCA1* by using droplet digital PCR (ddPCR) analysis. According to previous reports, individuals with BRCA1 abnormalities had an approximately 80% higher risk of developing breast cancer.^39^^,^^40^ *BRCA1* overlapped-large gene deletion was associated with a 1.21-fold elevated breast cancer risk.^41^ In line with our results, the microtumor and tissue-pairs of BRC17, BRC21, and BRC17-2 cases were correlated together with the observed copy number losses (CNA values < 2). Only the BRC19 case presented copy number gain (CNA value > 2), although the CNA value of tissue was due to the negative cut-off (the cut-off value = 2 was referred to from previous studies^13^^,^^42^^,^^43^). This was possibly related to the higher proportion of non-malignant cells in mastectomy-derived tissue, when compared to the matched PDMs. Related to the previous studies,^13^^,^^44^ all cases demonstrated that the CNA values of PDMs likely exhibited more distinct abnormalities than the primary tissues (Figure 5C).

### Breast cancer patient-derived microtumors as a proof-of-concept for predicting neoadjuvant chemotherapeutic responses

To prove that breast cancer PDMs were feasible to utilize as a therapeutic prediction model regarding clinical turnaround time, the PDMs were tested with preferred neoadjuvant chemotherapy (paclitaxel and doxorubicin)^7^^,^^45^^,^^46^ in parallel with observing patient outcomes. The successful cases of microtumor generation were selected to perform drug screening following criteria that were already validated by histological and genetic analyses (Figure 5) and required an availability of more than or equal to 15 Matrigel domes. Also, the individual domes needed to consist of at least 5 active microtumors with diameters less than 200 μm^17^^,^^18^^,^^47^^,^^48^ and capable tracking of their growth rates. Based on the criteria, the mechanically isolated PDMs of BRC17 and BRC19 cases and the enzymatically isolated PDMs of BRC21 and BRC17-2 cases were included. Briefly, the microtumors were cultured in 96-well culture plates after tissue isolation for 1 to 2 weeks, and then they were exposed to chemotherapy for 144 h (or 6 days) prior to endpoint analyses. To evaluate drug efficacy, the treated PDMs were stained with Calcein AM and BOBO-3 iodide to visualize live- and dead-signal areas. Subsequently, the individual PDMs were measured for signal areas, and the percentage relative portion of live area was calculated according to an equation from the previous study^17^ in the STAR Methods (microtumor viability analysis). Half-maximal inhibitory concentration (IC50) and %normalized area under the dose-response curve (AUC) values were identified.^18^^,^^49^^,^^50^^,^^51^ According to the previous report,^50^ microtumor drug responses were determined by readout %AUC into 2 groups, including chemosensitivity (AUC < 50%) and chemoresistance (AUC ≥ 50%), prior to correlating with clinical outcomes.

For doxorubicin, the PDMs from BRC17, BRC19, and BRC21 cases presented the decrease of the percentage relative portion of live area following a dose-dependent manner (the IC50 values of 26.72 μM, 13.93 μM, and 40.94 μM respectively), although BRC17 and BRC21 cases still exhibited the 48.33% and 49.31% of live areas at the highest dose. In contrast, the PDMs from BRC17-2 case demonstrated no significant changes in the percentage relative portion of live area in a dose-dependent manner (the IC50 value rather than 100 μM) (Figures 6A, 6C, and S1). Subsequently, these PDM cases were classified into sensitive and resistant groups based on %normalized AUC values.^50^ The PDMs from BRC17 and BRC17-2 cases, which were derived from the same recurrent patient at different times and surgical types, had concordance in categorized resistance with the AUC values of 51.28% and 57.33%. The PDMs from the BRC21 case were also resistant to doxorubicin, with AUC values of 50.90%. On the other hand, the PDMs from the BRC19 case were sensitive to the drug, with AUC values of 45.22% (Figure 6B). When assessed these interpretations with a medical history, the matched patient of BRC17 and BRC17-2 cases was interesting. In 2012, invasive ductal carcinoma (IDC) with mixed mucinous features was first diagnosed prior to being treated with doxorubicin. Following 7-year treatment, both similar histological characteristics and the right-breast site of the relapsed tumor were detected by ultrasound. Thus, the primary tumor was likely not solely sensitive or resistant to doxorubicin (Figure 6D). In correlation, the BRC17 and BRC17-2 microtumors were interpreted in the context of resistance to doxorubicin, provided a close reflection of the patient outcomes.

**Figure 6:**
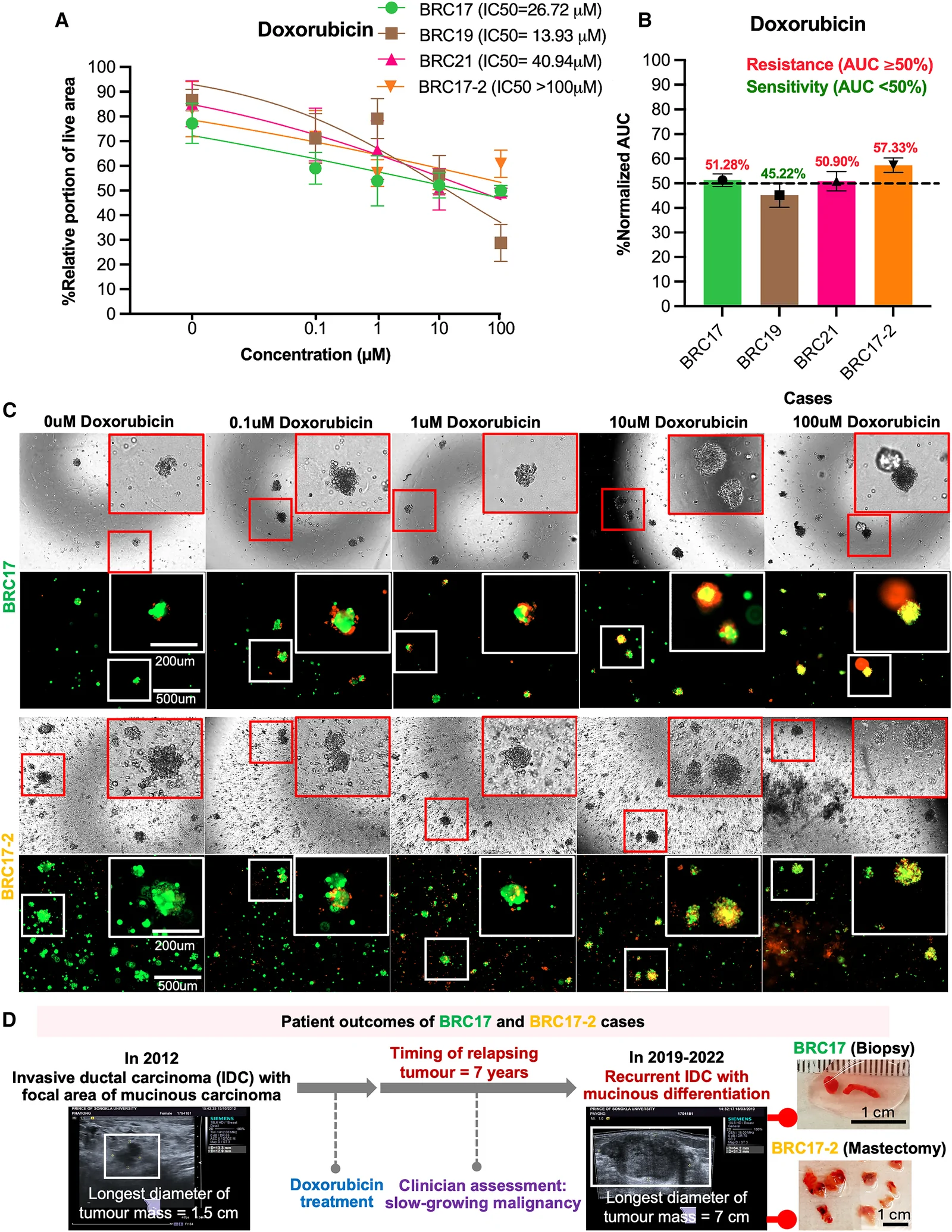
Doxorubicin response efficacies of breast cancer PDMs and the clinical relevance between the BRC17- and BRC17-2-PDMs and the matched patient (A) The percentage relative portions of live areas of breast cancer microtumors after exposure to doxorubicin for 144 h (mean ± SD from 5 to 19 analyzed microtumors per condition for BRC17, BRC21, and BRC17-2 cases, and from 6 to 7 analyzed fields per condition from images with a size of 1305∗1305 μm square from whole well for BRC19 case). The IC50 values of the 4 analyzed cases were presented in the graph labeling. (B) %Normalized the area under the dose-response curve (AUC) values in each case were demonstrated as mean ± SD, and the individual cases were interpreted microtumor drug efficacies by classifying as sensitivity (AUC <50%) and resistance (AUC ≥ 50%). The 95% confidence interval ranges (95%CI) of BRC17, BRC19, BRC21, and BRC17-2 were 46.28–56.27%, 35.63 to 54.81%, 43.19 to 58.60%, and 51.48 to 63.18%, respectively. (C) The fluorescent images of Calcein AM (live indicator in green color) and BOBO-3 iodide (dead indicator in red color) staining displayed the microtumor viability after doxorubicin exposure for 144 h (each condition containing at least 5 active microtumors per well, enabling tracking growth rates along the growing period, and the results of total 4 analyzed cases were presented in Figure S1; scale bars, 500 μm and 200 μm for the low- and high-magnification images respectively). (D) The medical history of BRC17 and BRC17-2 cases, in which the tissue specimens were collected from the same patient at different times and with different surgical types, indicated the recurrence of the same cancer characteristics after 7-year doxorubicin treatment. For the tumor behavior, the clinicians assessed the patient’s ultrasound tracking and reported a slow-growing malignancy.

In another drug, the PDMs from BRC17, BRC19, BRC21, and BRC17-2 cases demonstrated a decrease in the percentage relative portion of live area in a dose-dependent manner, with IC50 values of 0.51, 1.61, 3.4, and 1.59 μM (Figures 7A, 7C, and S2). Referring to the AUC assessment, these PDM cases were classified in the susceptible group with the AUC values of 28.33%, 38.21%, 32.87%, and 25.15%, respectively (Figure 7B). In line with a medical report, the microtumors from the BRC21 case were established from a core needle biopsy (as small as ∼0.1 × 0.1 × 1.3 cm^3^) of the locally advanced patient who was classified as BI-RADS 6 or biopsy-proven malignancy. The patient was treated with paclitaxel and then was considered to have a complete response after 6 months, based on physical examination, magnetic resonance imaging (MRI), and histopathological analysis. Clinicians reported that the tumor mass decreased by approximately 48% in its longest diameter after treatment and was appropriate to remove with Modified Radical Mastectomy (MRM) (Figure 7D). Ultimately, the patient showed no evidence of both residual tumor and extranodal extension following the surgery and successfully recovered. Concordantly, the AUC interpretation of BRC21-microtumors also well responded to paclitaxel treatment.

**Figure 7:**
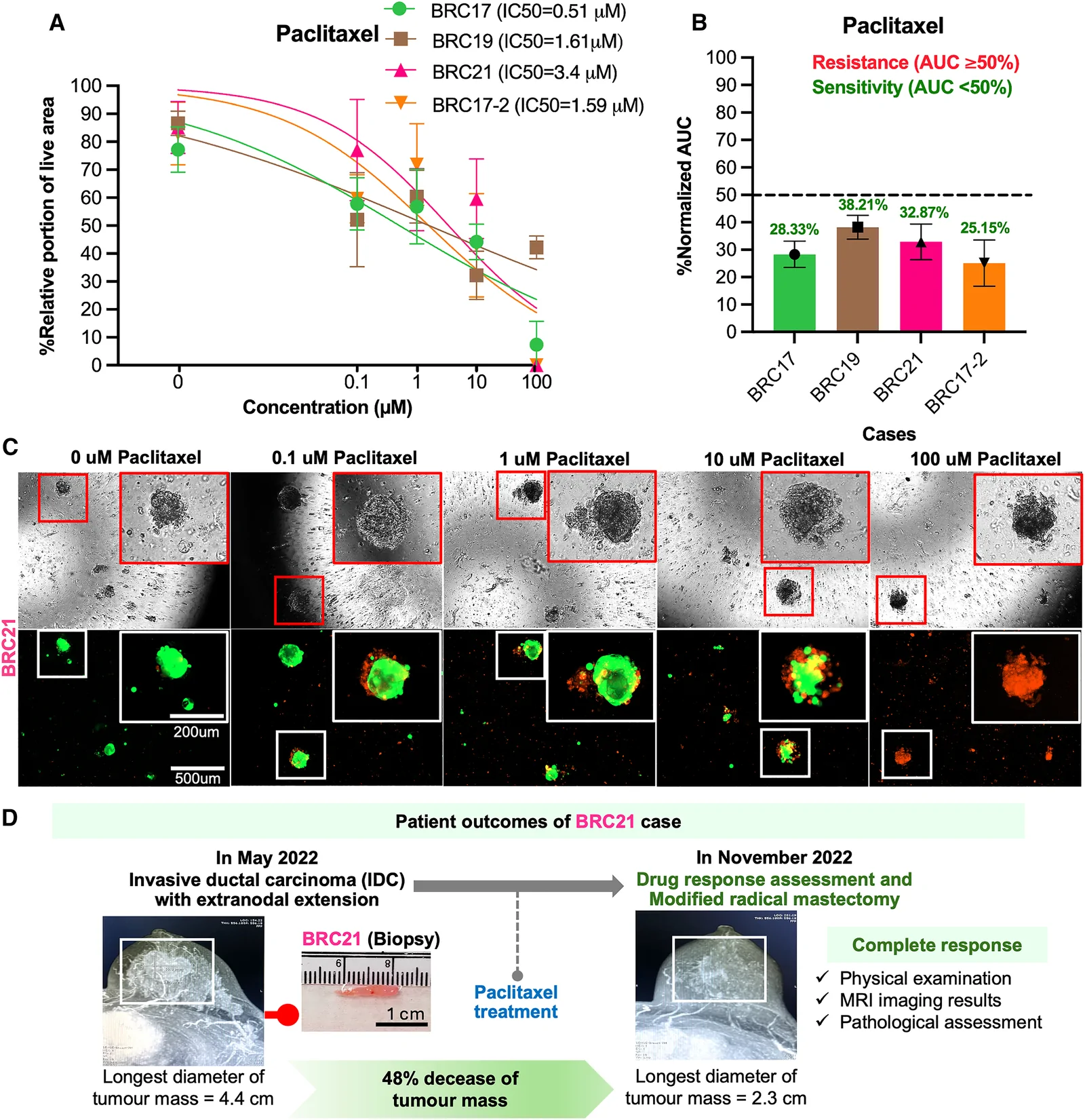
Paclitaxel response efficacy of breast cancer PDMs and the clinical relevance of the BRC21-PDMs and the matched patient (A) The percentage relative portions of live areas of breast cancer microtumors after exposure to paclitaxel for 144 h (mean ± SD from 5 to 19 analyzed microtumors per condition for BRC17, BRC21, and BRC17-2 cases, and from 6 to 7 analyzed fields per condition from images with a size of 1305∗1305 μm square from whole well for BRC19 case). The IC50 values of the 4 analyzed cases were presented in the graph labeling. (B) %Normalized the area under the dose-response curve (AUC) values in each case were demonstrated as mean ± SD, and the individual cases were interpreted microtumor drug efficacies by classifying as sensitivity (AUC <50%) and resistance (AUC ≥ 50%). The 95% confidence interval ranges (95%CI) of BRC17, BRC19, BRC21, BRC17-2 were 18.98–37.68%, 29.66 to 46.77%, 20.17 to 45.57%, 8.67 to 41.64%, respectively. (C) The fluorescent images of Calcein AM (live indicator in green color) and BOBO-3 iodide (dead indicator in red color) staining presented the microtumor sensitivity to paclitaxel after 144h treatment (each condition containing at least 5 active microtumors per well, enabling tracking growth rates along the growing period, and the results of total 4 analyzed cases were presented in Figure S2; scale bars, 500 μm and 200 μm for the low- and high-magnification images respectively). (D) The patient in the BRC21 case was diagnosed with locally advanced-stage breast cancer, and a core needle biopsy was collected for PDM development and drug testing in parallel with clinical paclitaxel treatment. After that, the patient was assessed through physical examination, MRI imaging, and pathological analysis, resulting in a complete response.

Taken together, breast cancer patient-derived microtumors were feasible to utilize as therapy prediction models, offering a turnaround time of 2–3 weeks after surgery (Figure 8A). The microtumors from individual cases could exhibit variable responses to chemotherapy. Referring to overall results, all microtumor cases were likely less sensitive to doxorubicin than paclitaxel (Figures 8B and 8C). Besides, the microtumors had the potential to appear consistent with the patient outcomes, achieving a 100% concordance (Figure 8D).

**Figure 8:**
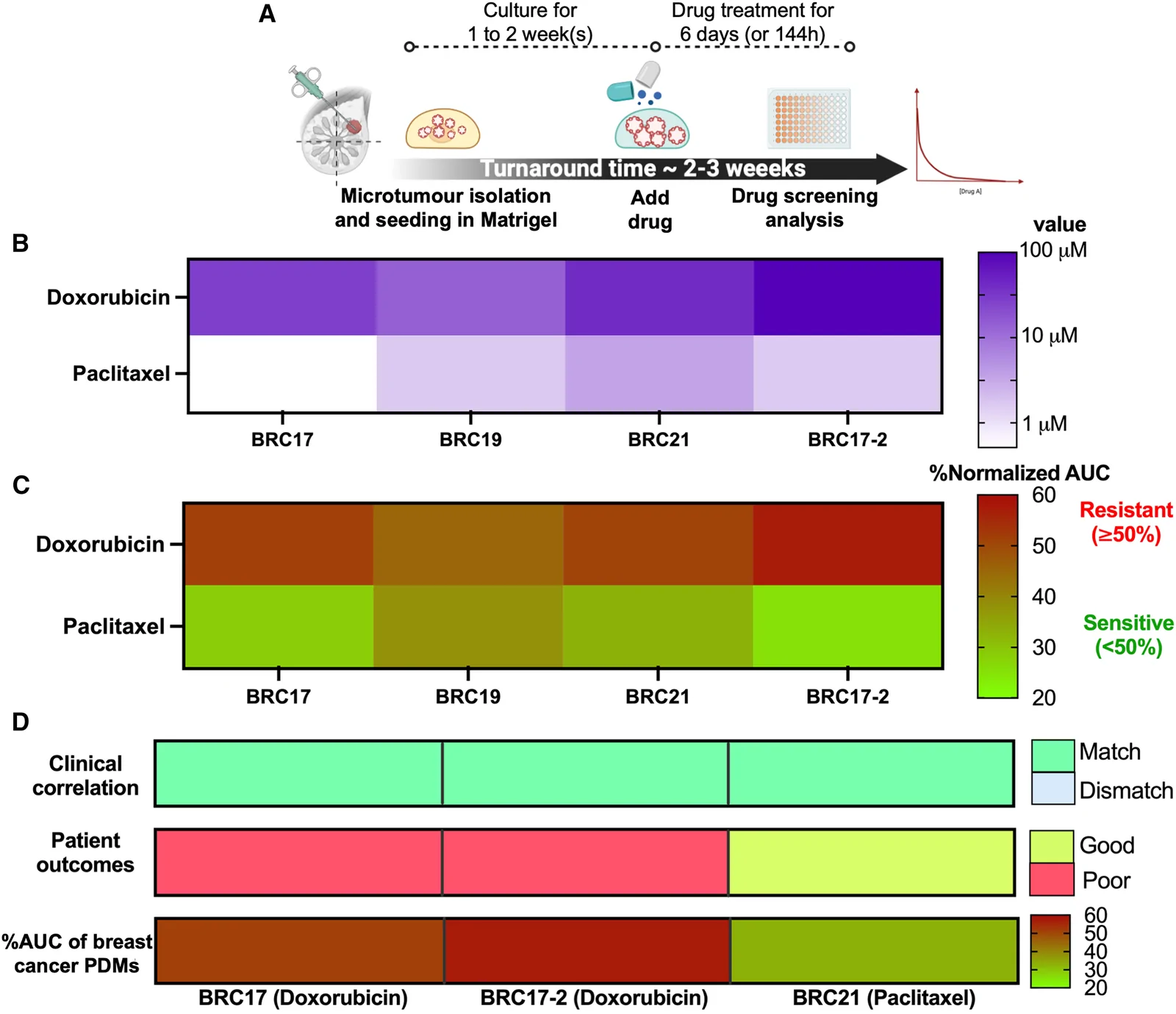
Breast cancer PDM platform for potential use in chemotherapeutic response predictions (A) The schematic image showed a drug screening timeline from surgery to drug response prediction with a turnaround time of about 2–3 weeks. (B and C) The heatmaps of IC50 values and %normalized AUC of two neoadjuvant chemotherapeutic agents in all cases were demonstrated. (D) The heatmaps summarized the concordance of the patient outcomes and the %AUC of microtumors, interpreted by sensitivity (AUC <50%) and resistance (AUC ≥ 50%). Briefly, the PDMs of BRC17 and BRC17-2 cases, derived from the same patient, had resistance to doxorubicin consistent with the patient’s cancer relapse. In addition, the PDMs of the BRC21 case were highly sensitive to paclitaxel, corresponding to the patient’s complete response. Overall, this achieved a 100% concordance (*N* = 3 cases).

## Discussion

Breast cancer organoids have emerged as a promising therapy prediction model with the potential to reflect patient outcomes.^13^^,^^15^^,^^16^^,^^17^^,^^18^^,^^52^ Nevertheless, the low yields of small tissue specimens and the high cost of niche factor-enriched medium remained important challenges obstructing clinical translation.^16^^,^^18^^,^^25^ Therefore, our study aimed to improve an effective culture protocol and to optimize a cost-effective culture medium for properly establishing breast cancer patient-derived microtumors (PDMs), sourced from core needle biopsies and small tissue specimens. The generated PDMs were utilized to screen neoadjuvant chemotherapy in parallel to the matched patients for validating a personalized predictive application.

The interpretation of establishment efficiency in tumor biopsy-derived cultures nonetheless warrants caution. It has been reported that, in dissociated-, re-aggregated-, and serially passaged-organoid cultures, non-malignant epithelium may outgrow the tumor compartment, with tumor-specific alterations falling below detection within early weeks.^26^ Our approach differs mechanistically: PDMs were maintained as intact fragments without complete dissociation or passaging process, such that the normal single-cell proportion was largely excluded during isolation and clonal competition between normal and malignant cells was avoided. Moreover, the tumor authenticity of PDMs was confirmed by integrating multi-platform approaches for orthogonal pathomolecular characterization and authentication rather than inferred from single-method validation. Notably, short-tandem repeat (STR) profiling mostly demonstrated 100% identity to the matched tumor tissues, and *BRCA1* copy-number alterations were more pronounced abnormalities in the PDMs than in the corresponding tissues. This indicated enrichment rather than attenuation of the tumor compartment across the assay window. Accordingly, we framed our study as the efficient establishment of ‘microtumor or *ex vivo* tumor fragment cultures’^27^^,^^28^^,^^29^ from core-needle biopsies and small tissue specimens for drug screening within a clinically relevant turnaround time, rather than conventional organoid improvements.

By adapting a previously published culture protocol and FBS-based medium recipe from our research team,^53^ in this study, we develop a simple technique and two optimal steps to suitably generate breast cancer PDMs from core-needle biopsies and small tissue specimens. Interestingly, the biopsy volume as small as ∼0.1 × 0.1 × 0.7 cm^3^ (BRC17 case) and a single biopsy specimen (BRC21 case) were successfully utilized for microtumor establishment, molecular validations, and neoadjuvant chemotherapy screening. Referring to our modified protocol, observing tumor fragment and cell cluster release during the mincing step was initially developed as a simple technique for evaluating the chance of PDM derivation. This phenomenon was detected in most successful cases for PDM generation but was absent in failure cases. Because of the quality of tissue specimens affecting PDM yields,^54^^,^^55^^,^^56^ we further investigated the histological characteristics of original tissues with H and E staining. In accordance with the observations, the tissue specimens that successfully yielded microtumours exhibited a higher number of cancer epithelial cells.

To enhance PDM derivation success, our modified protocol suggested collecting tumor fragments from both mechanical and enzymatic isolations and culturing them in separate batches. Fundamentally, breast cancer development predominantly induced lipid metabolism and adipogenesis,^57^^,^^58^ and this resulted in lower tissue stiffness than other solid cancer types, which were pivotal in fibroblast accumulation (such as prostate cancer^59^). Based on this rationale, variations in fragment yields between mechanical and enzymatic isolations were observed, and half of all successful cases demonstrated higher yields via mechanical isolation. Notably, in comparison between mechanical and enzymatic isolations, the PDMs exhibited a resemblance in proliferation profiles, as indicated by their morphological phenotypes and growth rates. In another, FBS-based culture medium was refined from our previously published recipe^53^ to be appropriately utilize for breast cancer PDM establishment. The supplement of the ROCK pathway inhibitor (or Y-27632) was an important strategy to improve the FBS-based medium for sustaining breast cancer microtumor growth, the expression of microtumor phenotypes, and survivability, as shown in Figure 2.

In agreement with the previous studies, Y-27632 was indicated as an essential component to promote cell proliferation and anti-apoptosis for breast cancer organoid culture,^13^^,^^35^^,^^60^ particularly in a serum-based medium.^22^ In both ER-positive and ER-negative subtypes of breast cancer, the organoids required Y-27632 exposure to effectively maintain their growth for over half a month after isolation.^35^ In addition, the Y-27632 supplement could induce the expression of cystic and hollow phenotypes better than the control. In our findings, which were obtained from triple-negative PDMs (BRC19 case), we reviewed the molecular mechanism through which this cancer subtype generally exhibited RhoA/ROCK overexpression, related to migratory behaviors and poor prognosis.^61^^,^^62^ In contrast, ROCK inhibition significantly induced triple-negative breast cancer tumortumouroids displaying a more organized morphological phenotype, due to reduced actomyosin contractility and restored basal polarity.^63^ Collectively, FBS-based medium with Y-27632 addition not only supported breast cancer PDM growth but also enhanced the formation of cystic and hollow microtumors better than the absent one. Besides, the supplemented medium demonstrated the capacity to sustain high living cell density within microtumors despite culturing for over 2 to 3 weeks.

As for other potential factors supporting breast cancer PDM growth, FBS served as a nutrient-enriched component and supported inducing breast cancer cells into S-phase entry.^64^^,^^65^^,^^66^ Focusing on endogenous estradiol (E2) present, containing about 2.23 pg/mL in 12%FBS, it was reported as a key hormone to enhance breast cancer cell proliferation.^65^^,^^67^ For the ER-positive subtype, the E2/ER signaling pathway involves activating the transcription of several oncogenes.^67^^,^^68^^,^^69^ For instance, the serum-derived E2 could stimulate the expression of the c-MYC oncoprotein, acting as the master regulator of breast cancer outgrowth.^68^^,^^70^ For the ER-negative subtype, the hormone was still necessary to enhance the healthy morphology and growth capacity of breast cancer cells^67^ through E2 binding to the G protein-coupled estrogen receptor (GPER) and triggering multiple signaling cascades. The downstream signaling included EGFR/Calcium-signaling pathways for driving pro-tumorigenic niches and the cAMP signaling pathway for activating cAMP response element-mediated cell cycle transcriptions.^71^^,^^72^^,^^73^ Furthermore, when concentrated on Gibco-sourced FBS (Ref. 10270–106), there was evidence that it could sustain proliferative malignant cells and induce cellular IL-8 release, acting as dual autocrine/paracrine growth factors for driving breast cancer progression and against anoikis.^74^^,^^75^^,^^76^

Through in-depth validations, breast cancer PDMs were verified for their capacity to mimic original tumor heterogeneity. Firstly, based on proliferation analysis, we revealed that overall growth rates were significantly higher in aggressive cancer subtypes, related to the previous report.^77^ There were the lowest growth rates for ER-positive-derived PDMs (BRC16, BRC17, and BRC17-2 cases) and the highest growth rates for triple negative- and HER2-positive-derived PDMs (BRC19, BRC20, and BRC21 cases). Remarkably, BRC17 and BRC17-2 PDMs, derived from the same patient at different times and surgical types, demonstrated comparably low proliferation rates, reflecting the slow-growing malignancy of the *in vivo* patient tumor. The slow growth patterns of BRC17 and BRC17-2 PDMs also reflected the characteristics of IDC mixed mucinous, which typically exhibited extremely low proliferative activity and low mitotic rate.^78^^,^^79^

Next, we investigated the histopathological characteristics of the generated PDMs, derived from mechanical and enzymatic isolations, in comparison to the matched tissues. The PDMs from both isolation methods could maintain the appearance of typical carcinoma histological features, respective to the tumor origins. For instance, the triple negative-derived PDMs (BRC19 case) exhibited signet ring-like cells and mitotic figures which were related to an aggressive stage and a poor prognosis,^80^^,^^81^ as resembling the patient outcome. In addition, the expression of microtumor phenotypes generally agreed with the histological characteristics of tissue origins. We observed that the generated microtumors predominantly displayed a solid and dense phenotype with or without the mixing of other forms. Consistent with the previous report,^13^ ductal carcinoma specimens often gave rise to coherent organoid. Furthermore, the immunohistochemical analysis of ER/PR/HER2 expression verified that the PDMs, derived from mechanical- and enzymatic isolations, exhibited high similarity in correlation with their matched tissues.

Upon genetic analysis, multilocus STR profiling proved that most of the PDM-tissue cases demonstrated a 100% identical genetic profile. Although BRC21 case showed an 80% probability with most of single-step mutations, this was still higher than the cut-off value of 75% for the concordance rate,^82^ implied genetic homology. In addition, we noted that germline STR loci are shared by all cells of an individual, so the STR concordances indicated the same-patient origins of tissue-PDM pairs rather than tumor identity. As this reason, the malignant compartment of generated PDMs was instead confirmed by histological and immunohistochemical features (Figure 5A) and the retention of tumor-associated *BRCA1* copy-number alterations (Figure 5C). The single-step mutations might occur from cancer mosaicism and intratumoral heterogeneity, affecting genetic variation within the same batch.^38^ Besides, the PDM-tissue cases were validated with ddPCR to verify the CNA profiles of *BRCA1*. This comparison depicted 100% similarity across all cases, regarding both copy number loss and gain. In terms of the BRC19 case, which was derived from a triple-negative mastectomy, in spite of finding copy number gain in both the microtumours and the matched tissue, the value of CNAs in the tissue was slightly higher than the negative cut-off. Otherwise, the value of CNAs in the generated microtumors clearly demonstrated copy number gain. In line with the previous reports,^13^^,^^44^ the CNA signals of microtumors were more distinct than tissues, associated with a higher purity of tumor cell portion and lower contamination by non-malignant cells. With regard to this, all PDM cases in our study exhibited more abnormalities than the matched tissues in both copy number loss and gain. Alternatively, triple negative-derived PDMs, were previously reported^44^ the least concordance of CNA profiles as the marked variability of cell subpopulations, such as hyperoxia-sensitive subclone.

As a final validation, breast cancer PDMs were tested to determine whether they could preserve their matched patients' neoadjuvant chemotherapy responses and could offer utility as therapeutic prediction models within clinical turnaround time. In parallel to clinical treatments, the PDMs were treated with doxorubicin and paclitaxel, referred from the preferred regimens of neoadjuvant chemotherapy.^7^ Because our modified culture protocol primarily established PDMs with a fragment-based culture, the heterogeneity of sizes, densities, and morphological phenotypes was inevitable. Here, the percentage relative portion of live area which was calculated from the live/dead imaging of individual microtumors was utilized as a main approach for evaluating treatment efficiencies, following the previous study.^17^ The ratios of live- and dead-signal areas in individual microtumours were analyzed, thereby facilitating the reduction of the influence of heterogeneous microtumour formations within the same Matrigel dome. Also, the live/dead quantification capably overcame the limitations of traditional viability assays (e.g., CellTiter-Glo) that total live signals from individual whole wells could not be discriminated between breast cancer microtumors and contaminating normal cells.^17^ Furthermore, to interpret PDM drug efficacies, the area under the drug response curve (AUC) was a critical response metric that was utilized in this study for classifying the treated PDMs into a sensitive group (AUC < 50%) and a resistant group (AUC ≥ 50%). The AUC metric was reported as having the highest predictive performance because it combines drug efficacy and potency into a single value of %normalized AUC in individual cases.^83^ In terms of reflecting patient outcomes, there was interesting evidence that the AUC metric effectively translated organoid therapeutic results with over 90% accuracy.^50^

With respect to our results, all PDM cases responded to doxorubicin, ranging from sensitivity to resistance, and were consistently sensitive to paclitaxel, based on the interpretation of AUC values. When validated with actual clinical outcomes, the PDMs had a positive correlation with patient responses. In the case of chemosensitivity prediction, BRC21-PDMs and the matched patient were consistent after paclitaxel treatment. While the PDMs were susceptible to the drug with an AUC value of 32.87%, the patient was diagnosed by clinicians with a complete response to neoadjuvant paclitaxel treatment, based on physical examination, MRI imaging, and pathological analysis. Eventually, the patient underwent mastectomy for tumor removal and recovery. Conversely, in the case of chemoresistance prediction, BRC17 and BRC17-2 PDMs, derived from the same patient at different times and surgical types, closely resembled the actual patient relapse outcome following doxorubicin treatment. Whereas these PDM cases were classified as resistance to the drug with AUC values of 51.28% and 57.33%, the matched patient had a recurrence with a similar histological type and breast site after 7 years of receiving doxorubicin. Therefore, the resistant PDMs from BRC17 and BRC17-2 cases confirmed each other, aligning with the patient’s ultrasound-monitored tumor regrowth.

Collectively, these findings position the present platform within the patient-derived microtumor, rather than the organoid, paradigm. Accordingly, we frame our contribution as the efficient establishment of tumor-validated microtumors (*ex vivo* tumor fragments) from core-needle biopsies and small tissue specimens for functional drug screening within a clinically relevant turnaround time, rather than as an improvement in organoid derivation. This distinction is important. A recent study^26^ reported that, in an early-stage needle-biopsy setting, more than 85% of breast cancer organoid cultures were overtaken by normal-cell overgrowth, with tumor-specific alterations lost within approximately 2–3 weeks and tumor organoids obtained predominantly from more advanced, mastectomy-derived specimens. By maintaining intact tumor fragments without dissociation or serial passaging, our approach may reduce the selective competition between normal and malignant epithelial populations that can occur during prolonged organoid culture. Across the short-term assay window, sample identity was maintained, as demonstrated by 100% STR concordance, together with retention of tumor-associated *BRCA1* copy-number alterations. Thus, the fragment-based PDM approach provides a practical alternative for functional testing of small biopsy specimens in settings where conventional tumor organoid establishment remains challenging.

Within the microtumor paradigm, our platform closely parallels previously reported breast cancer microtumor models,^27^^,^^28^ in which intact tumor fragments are maintained in short-term culture (∼1–2 weeks) without serial passaging, retain components of the native tumor microenvironment, and are used for functional drug testing. However, our tissue-processing workflow differs in that PDM-sized fragments were retained at both stages of tissue processing: fragments released during mechanical dissociation were collected for culture, while the remaining tissue underwent enzymatic digestion to recover additional, including smaller, microtumor fragments. This dual-stage fragment recovery strategy was designed to maximize the utilization of limited tumor material, which is particularly relevant for core-needle biopsies and other small tissue specimens Our study further extends the microtumor concept to the neoadjuvant setting by establishing PDMs from core-needle biopsies rather than predominantly surgical specimens, maintaining them in a simple and relatively cost-effective FBS-based medium supplemented with Y-27632 rather than a defined serum-free medium^27^^,^^28^ or a niche-factor-enriched organoid medium,^13^ and relating *ex vivo* drug responses to patients’ actual neoadjuvant treatment outcomes.

An additional potential advantage of the fragment-based PDM format is the preservation of components of the native tumor microenvironment. Previous breast cancer microtumor studies have demonstrated retention of fibroblast activation protein-α (FAP-α)-positive fibroblasts,^28^ while tissue aggregate-derived tumoroids have been reported to retain α-smooth muscle actin (α-SMA) and vimentin-positive stromal cells, in contrast to models generated from dissociated cell suspensions.^33^ These observations suggest that fragment-based culture formats may better preserve stromal compartments, including cancer-associated fibroblasts (CAFs), during short-term PDM culture.

Taken together, our study establishes a practical culture workflow and an optimized FBS-based medium for generating breast cancer PDMs from core-needle biopsies and small tissue specimens. The platform enabled pathomolecular validation and short-term functional drug testing within approximately 2–3 weeks of tissue acquisition. This interval falls within the clinically relevant period of approximately 28–35 days between diagnostic biopsy and initiation of neoadjuvant therapy reported in previous studies,^23^^,^^24^ supporting the feasibility of integrating *ex vivo* PDM testing into the treatment-planning window. These findings provide a foundation for prospective clinical validation of the platform.

### Limitations of the study

The present study still has several limitations. First, in future studies, a larger sample size with diverse populations and more tailored anti-cancer drug regimens should be used to yield more accurate, reliable, and generalizable data for clinical use. Second, the molecular pathways and bioactive components of FBS-based medium should be further clarified through proteomic and mechanistic analyses of both within-lot and between-lot variations. This would help to improve PDM culture standardization and reproducibility as well as to facilitate future scale-up of a living biobank of breast cancer microtumors. Third, CAFs and other stromal populations were not specifically immunophenotyped, limiting conclusions regarding tumor-microenvironment preservation and its contribution to drug response. Finally, the current workflow was optimized for short-term functional testing and requires further validation for long-term expansion or generation of stable microtumor cultures. In addition, all participants were female, so the influence of sex or gender on the results could not be evaluated. Addressing these limitations will be important for prospective clinical translation.

## Resource availability

### Lead contact

Requests for resources, reagents, or additional information should be directed to the lead contact, Dr. Pasarat Khongkow (pasarat.k@psu.ac.th; k.pasarat@gmail.com).

### Materials availability

Patient-derived microtumors (PDMs) and cryopreserved stocks generated in this study are available from the lead contact upon reasonable request, subject to approval by the Human Research Ethics Committee of the Faculty of Medicine, Prince of Songkla University, and completion of an appropriate material transfer agreement (MTA).

### Data and code availability

• De-identified quantitative data and original microscopy images reported in this paper are available from the lead contact upon reasonable request. Individual-level clinical data are not publicly available to protect participant privacy and comply with the Thai Personal Data Protection Act.
• This paper does not report original code.
• No standardized datasets requiring public repository deposition were generated; therefore, no accession numbers apply.
• Additional information required for data reanalysis is available from the lead contact upon reasonable request.

## Acknowledgments

We would like to thank all patient participants and clinicians for consents and tissue specimens. This research project was supported by the National Research Council of Thailand (NRCT): Royal Golden Jubilee PhD scholarship (NRCT5-RGJ63019-161). In addition, we thank the Department of Biomedical Sciences and Biomedical Engineering, the Department of Surgery, the Department of Pathology, and the Translational Medicine Research Center in the Faculty of Medicine at 10.13039/501100004508Prince of Songkla University for supporting facilities and information to do this research. We would like to thank the Office of Human Research Ethics Committee (HREC), the Faculty of Medicine, Prince of Songkla University (REC.62-234-5-2) for suggestion and approving patient informed consents.

## Author contributions

Conceptualization, P.K., S.L., P.W., and M.P.; investigation and data analysis, P.K., M.P., S.L., P.W., P.T., and P.K.; methodology, P.K., M.P., S.L., P.W., P.T., P.K., R.N., O.P., and K.U.; writing – original draft preparation, P.K., M.P. and K.U.; review and editing, P.K., and M.P; all authors have read and agreed to the published version of the manuscript.

## Declaration of interests

The authors declare no competing interests.

## STAR★Methods

### Key resources table

**Table:** 

REAGENT or RESOURCE	SOURCE	IDENTIFIER
**Antibodies**		

Estrogen receptor rabbit monoclonal antibody (Clone SP1)	Epredia	Cat# RM-9101-S; RRID: AB_149901
Mouse monoclonal antibody progesterone receptor	Leica	Cat# NCL-L-PGR-312; RRID: AB_563967
Anti-HER-2/neu (4B5) rabbit monoclonal primary antibody IVD	Ventana	Cat# 790–2991; RRID: AB_2335975

**Biological samples**		

Human breast cancer tissues, derived from core needle biopsies and mall tissue specimens	This study	Tables 1, S1, and S2

**Chemicals, peptides, and recombinant proteins**		

DMEM, high glucose, pyruvate	Gibco	11995–065
Ham’s F-12 Nutrient Mix	Gibco	11765–054
Penicillin-Streptomycin	Gibco	15140–122
HEPES	Gibco	15630–080
GlutaMAX™ Supplement	Gibco	35050–061
Primocin	Invivogen	ant-pm-1
Y-27632 (Dihydrochloride)	Stem cell technologies	72304
Fetal Bovine Serum, qualified, Brazil	Gibco	10270–106
Neutral buffered formalin solution	Sigma Aldrich	HT501128
Collagenase, Type II, powder	Gibco	17101–015
Matrigel® Growth Factor Reduced (GFR) Basement Membrane Matrix, Phenol Red-free, LDEV-free	Corning	356231
Recovery™ Cell Culture Freezing Medium	Gibco	12648–010
Agarose	Vivantis technologies	PC0701
Paclitaxel injection USP (Intaxel)	Fresenius Kabi	1C 66/53
Doxorubicin hyarochloride injection USP (ADRIM)	Fresenius Kabi	1C 204/52

**Critical commercial assays**		

DNeasy Blood and Tissue Kits for DNA Isolation	Qiagen	69506
VeriFiler™Plus PCR Amplification Kit	ThermoFisher	A35495
ddPCR™ Copy Number Assay: BRCA1, Human, Homo sapiens	Bio-rad	dHsaCP2500367
LIVE/DEAD™ Cell Imaging Kit (488/570)	ThermoFisher	R37601

**Software and algorithms**		

BioTek Gen5 software for Imaging & Microscopy (version 3.16)	Agilent	N/A
Open-source ImageJ software (version 2.14.0/1.54f)	National Institutes of Health	N/A
GeneMapper™ software (version 1.6)	ThermoFisher	4475073
QuantaSoft™ software (version 1.7.4.0917)	Bio-rad	N/A
GraphPad Prism software (version 11.0.2)	GraphPad Software, Inc	N/A
Microsoft Excel software (version 16.107.4)	Microsoft	N/A

**Others**		

BioTek Lionheart FX Automated Microscope	Agilent	N/A
CKX41 Inverted Phase Contrast Microscope	Olympus	N/A
Definite Focus 3 microscope	Zeiss	N/A
3500 Genetic Analyzer for Human Identification	ThermoFisher	4406017
Automated Droplet Generator	Bio-rad	1864101
CFX 96 Touch Real-Time PCR Detection System	Bio-rad	N/A
QX200 Droplet Digital PCR System	Bio-rad	N/A

### Experimental model and study participant details

#### Derivation of breast cancer tissues and study approval

Eight tissue specimens were obtained from seven female patients of Thai ancestry who were classified according to the Breast Imaging Reporting and Data System (BI-RADS) as categories 4C–6.^84^ Six specimens were obtained by core needle biopsy and two by surgical resection at Songklanagarind Hospital, Thailand, between 2021 and 2022 (Tables 1, S1, and S2). All procedures were conducted in accordance with the Declaration of Helsinki and approved by the Human Research Ethics Committee of the Faculty of Medicine, Prince of Songkla University, Thailand (REC.62-234-5-2). All participants provided written informed consent. Clinical information, including age, medical history, diagnosis, and treatment response, was collected. All participants were female; therefore, the potential influence of sex or gender on the study findings could not be assessed. Patient-derived microtumours were established as primary cultures rather than established cell lines. Each culture was authenticated against its matched tumor tissue by short tandem repeat (STR) profiling, and routine mycoplasma testing was performed, with all tested cultures yielding negative results.

#### Breast cancer patient-derived microtumour (PDM) establishment

The tissues were preserved in a working medium (containing DMEM/F12 medium (1:1; 11995–065 and 11765–054, Gibco, USA), Penicillin/Streptomycin (1%*v/v*; 15140–122, Gibco, USA), HEPES (10 mM; 15630–080, Gibco, USA), Glutamax (1%*v/v*; 35050–061, Gibco, USA)) supplemented with Primocin (100 μg/mL; ant-pm-1, Invivogen, USA) on ice during the ∼0.5h–1h of transportation. Upon arrival, the tissues were washed and minced into 0.5 to 1 mm^3^ small pieces in a cold working medium. During this process, the chance of microtumour derivation could be estimated by the simple observation of releasing fragments and cell clusters into the medium under an inverted light microscope (Olympus, Japan). For genetic- and histological analyses, some tissue pieces were preserved at −80°C freezer and fixed in neutral buffered formalin solution (10%; HT501128, Sigma Aldrich, Singapore) respectively. After that, the suspension of remaining minced tissue and working medium were collected into a 15 mL-conical tube, sheared with a 1 mL-wide bore pipette tip, and subsequently gravity separated. While the supernatant which contained the fragments of mechanically isolated PDMs was collected, the minced tissue sediment was then processed by enzymatic digestion.

The tissue sediment was digested in a digestion solution (composed of a working medium supplemented with Collagenase type 2 (1 mg/mL; 17101–015, Gibco, USA) and Y-27632 (10 μM; 72304, Stem cell technologies, Canada)) for 15 to 30 min in a 140-rpm shaking incubator (Eppendorf, Germany). Then, the digest condition was inactivated with fetal bovine serum (FBS) (10%; 10270–106, Gibco, USA) and filtered by a 100 μm-cell strainer. On the strainer, the digested tissue was gently mashed with a 5 mL-syringe plugger to increase the yield of tumor fragments and subsequently rinsed it with a working medium. The filtrate was centrifuged to collect the fragments of enzymatically isolated PDMs at 500g for 5 min in 4°C.

The fragments of mechanically- and enzymatically isolated PDMs were separately mixed in growth factor-reduced Matrigel (90%; 356231, Corning, USA) which was diluted in a cold working medium. Subsequently, the 5 to 10 μL dome of the mixture was seeded in pre-warmed 96-well tissue culture plates. The individual domes composed of tumor fragments with the size range of less than or equal 100 μm in most of the population, about 10–30 fractions depended on their yields. Although the tumor fragments were primarily required for our modified protocol, the BRC19 case resulted in single cells after mechanical- and enzymatic digestions. So, we seeded the Matrigel domes which contained about 5,000 cells in each. The seeded plates were inverted and gotten solidification in a cell culture incubator for 10 min. Lastly, the domes were overlaid with the 120 μL of an optimized FBS-based culture medium (containing a working medium supplemented with FBS (12%, 10270–106, Gibco, USA), Y-27632 (5 μM; 72304, Stem cell technologies, Canada), and Primocin (100 μg/mL; ant-pm-1, Invivogen, USA). A single fetal bovine serum lot (10270–106, Gibco, USA) was used throughout to limit lot-to-lot variability.

For culture maintenance, culture medium was changed every 2 to 3 days during a 1 to 2-week expansion period. In some cases, the PDMs were preserved with a recovery cell culture freezing medium (12648–010, Gibco, USA) in nitrogen tanks for setting living biobank. To track the growth of individual PDMs, they were captured by Lionheart FX automated microscope (Agilent, USA) and inverted light microscope (Olympus, Tokyo, Japan) with z stack and/or montage modes. After that, the images were created by Z-projection and/or stitching, and analyzed by GEN5 software (version 3.16, Agilent, USA) and ImageJ software (version 2.14.0/1.54f) for measuring average diameter and area. The growth rates based on area at day 6 of individual PDMs was calculated following this equation^85^^,^^86^:

$$Individual\;PDM\;growth\;rates=\left[\frac{PDM\;area\;at\;day\;6-PDM\;area\;at\;day\;1}{PDM\;area\;at\;day\;1}\right]\times 100$$

### Method details

#### Histological and immunohistochemical analyses

Tissues and PDMs were fixed in neutral buffered formalin solution (10%; HT501128, Sigma Aldrich, Singapore) for 30 min. The fixed PDMs were embedded in agarose drop (2%; PC0701, Vivantis technologies, Malaysia). Then, they were processed by dehydration, embedding paraffin, microtome section (2 μm of thickness), hematoxylin and eosin (H and E) staining, and specific immunohistochemical staining. The specific biomarkers included estrogen receptor (ER) (1:250; RM-9101-S, Epredia, USA), progesterone receptor (PR) (1:2500; NCL-L-PGR-312, Leica, Germany) and human epidermal growth factor receptor 2 (HER2) (6 μg/mL; 790–2991, Ventana, Switzerland). All images were captured by Definite Focus 3 microscope (ZEISS, Germany).

#### Genetic analyses

Tissues and PDMs were isolated DNA yields by using DNeasy Kit (69506, Qiagen, Netherlands) following the manufacturer’s protocol. Concentration and purity were determined by Nanodrop (2000, Thermo Fisher Scientific, USA). To authenticate that each PDM culture originated from the same patient as its matched tissue, short tandem repeat (STR) analysis was performed on a total of 25 loci on different chromosomes, including Y Indel, D8S1179, D21S11, D7S820, CSF1PO, D3S1358, TH01, D13S317, D16S539, D2S1338, D19S433, vWA, TPOX, D18S51, D5S818, FGA, Amelogenin, Penta D, D22S1045, Penta E, D10S1248, D12S391, D1S1656, D2S441, and D6S1043. Amplification was processed using VeriFiler Plus PCR Kit (A35495, Thermo Fisher Scientific, USA) following the manufacturer’s instructions. The samples were run on 3500 Genetic Analyzer for DNA fragment analysis (4406017, Thermo Fisher Scientific, USA) and then were visualized by GeneMapper (version 1.6, 4475073, Thermo Fisher Scientific, USA).

Furthermore, the copy number alterations (CNAs) of *BRCA1* gene were analyzed by performing droplet digital polymerase chain reaction (ddPCR). The reaction composed of ddPCR master mix for probe (2×), assay for *BRCA1* (20×; FAM), assay for RPP30 (20×; HEX), Hae3 (5U), and DNA yield (referred to the assay ID of dHsaCP2500367; Bio-Rad, USA). It was adjusted volume to 25 μL by DNase free water and then was generated DNA-containing oil droplets by using a droplet generator (1864101, Bio-Rad, USA). The droplets were amplified by PCR (Bio-Rad, USA) using 1 cycle of activating enzyme at 95 °C for 10 min, denaturation at 94°C for 30 s and annealing at 60°C for 1 min total 40 cycles, 1 cycle of deactivating enzyme at 98°C for 10 min, and 1 cycle of cooling down at 4°C. Lastly, the copy number values of *BRCA1* were analyzed by QX200 Droplet Digital PCR (Bio-Rad, USA) and visualized by QuantaSoft (version 1.7.4.0917, Bio-Rad, USA).

#### *In vitro* drug screening

After growing breast cancer PDMs for 1 to 2 weeks, the cases, which were already validated by histological and genetic analyses, had the availability of more than or equal to 15 Matrigel domes, and individual domes containing at least 5 active microtumours, were selected. The active microtumours need to be capable of tracking their growth rates along expansion period and had mostly population in diameter size less than 200 μm.^17^^,^^18^^,^^47^^,^^48^ Subsequently, the breast cancer PDMs were treated with doxorubicin (1c 204/52, Fresenius Kabi, Germany) and paclitaxel (1c 66/53, Fresenius Kabi, Germany). Briefly, the old culture medium was replaced with the 120 μL of new culture medium (lacked Y-27632 and Primocin) with or without drugs in the concentrations of 0, 0.1, 1, 10, and 100 μM. The PDMs were exposed to the drugs for 144h prior to readout their drug efficacies. The PDMs were treated with different conditions in independent duplicate or triplicate drops, depending on their yields. Drug exposure was applied as a single continuous 144h treatment; the medium containing the relevant drug concentration was added once at the assay initiation, and no medium replacement or re-dosing was performed during the exposure.

#### Microtumour viability analysis

To readout drug efficacies, the PDMs were stained with LIVE/DEAD imaging Kit (R37601, Thermo Fisher Scientific, USA), followed by removing the old culture medium with or without drugs, adding Calcein AM/BOBO-3 (1:3 dilution in culture medium), and incubation for 20 min. The stained PDMs were captured by Lionheart FX microscope (Agilent, USA) with z stack and/or montage modes under GFP and Texas Red channels. Then, the images of individual PDMs were created Z-projection, stitched, merged the channels, and analyzed by GEN5 software (version 3.16, Agilent, USA) to quantify Calcein AM- and BOBO-3 iodide-signal areas (5–19 analyzed microtumours per condition for BRC17, BRC21, and BRC17-2 cases, and 6 to 7 analyzed fields per condition in size 1305∗1305 μm square from whole well for BRC19 case). The analyzed PDMs were filtered and selected in the average diameter size of equal or more than 30 μm to exclude single cells. In addition, minimum fluorescent intensity was set at equal or more than 10% to remove background fluorescence. The percentage relative portion of live area in individual PDMs was calculated by using the equation^17^:

$$\left[\frac{Calcein\;AM-signal\;area}{Calcein\;AM-signal\;area+BOBO-3\;iodide-signal\;area}\right]\times 100$$

### Quantification and statistical analysis

Statistical analyses were outlined in respective figure legends. All results were analyzed and visualized by Microsoft Excel software (version 16.107.4) and GraphPad Prism software (version 11.0.2, GraphPad Software, Inc., USA). Quantitative variable results were presented with mean ± SD and *p-*value, tested their statistically significant relations by using Welch’s unpaired *t* test method or ordinary one-way Anova with Tukey’s multiple comparison method. For the evaluation of microtumour drug efficacies, half-maximal inhibitory concentration (IC50) values were demonstrated with mean ± SD, and %normalized area under dose-response curve (AUC) values, following the trapezoidal rule, were presented with mean ± SD and 95% confidence interval (95%CI).^18^^,^^49^^,^^50^^,^^51^ The %normalized AUC values were utilized for interpreting microtumour responses to chemotherapeutic drugs, based on the classifications of sensitive cases (AUC values < 50%) and resistant cases (AUC values ≥ 50%).^50^
