# Supplementary material for: Core needle biopsy-derived breast cancer microtumors enable functional drug-response testing for precision oncology
Source: iScience. 2026 Oct 15;29(11):117914. doi: 10.1016/j.isci.2026.117914 (PMC13694900; doi:10.1016/j.isci.2026.117914)
Supplement: Supplementary file 1 [file mmc1.pdf]

iScience, Volume *29*

Supplemental information

Core needle biopsy-derived breast cancer microtumors enable functional drug-response testing for precision oncology

Mooktapa Plikomol, Suphawat Laohawiriyakamol, Piyanun Wangkulangkul, Paramee Thongsuksai, Pokphon Khirilak, Ratsamaporn Nontikarn, Onpawee Phanthuvet, Karan Ulhaka, and Pasarat Khongkow

SUPPLEMENTAL INFORMATION

Document S1. Figures S1 and S2 and Tables S1 and S2.

- **Figure S1.** Doxorubicin treatment of breast cancer PDMs, related to **Figure 6**
- **Figure S2.** Paclitaxel treatment of breast cancer PDMs, related to **Figure 7**
- **Table S1.** The characteristics of tissue specimens, related to **Table 1**
- **Table S2.** The clinical treatment of patient enrollments, related to **Table 1**

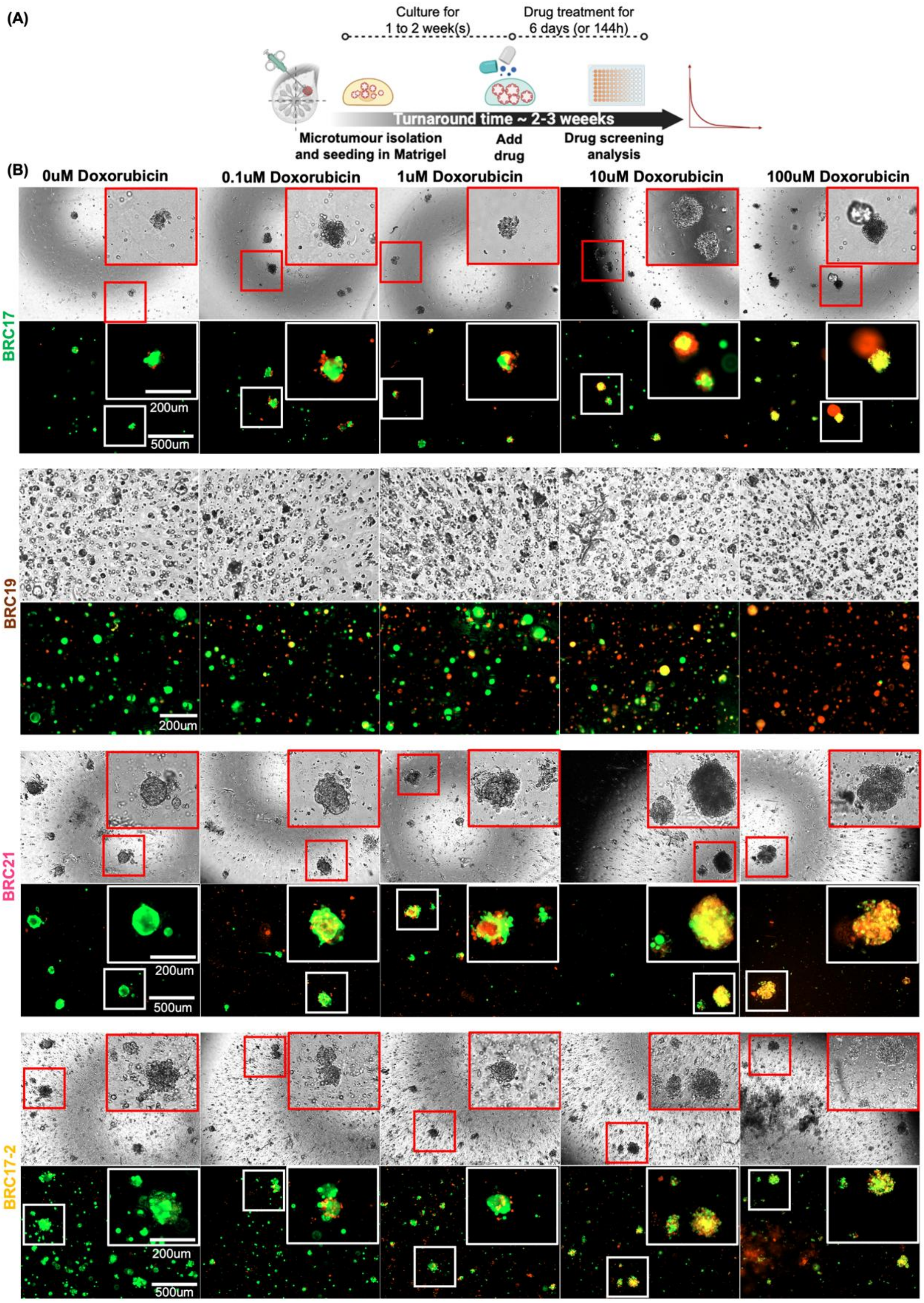

Figure S1. Doxorubicin treatment of breast cancer PDMs

(A) The schematic image showed in a drug screening timeline from surgery to analysis with a turnaround time about 2 to 3 weeks. (B) The fluorescent images of Calcein AM (live indicator in green color) and BOBO-3 iodide (dead indicator in red color) staining displayed the microtumour viability after doxorubicin exposure for 144 h (each condition containing at least 5 active microtumours per well, enabling tracking growth rates along the growing period, from total 4 analyzed cases).

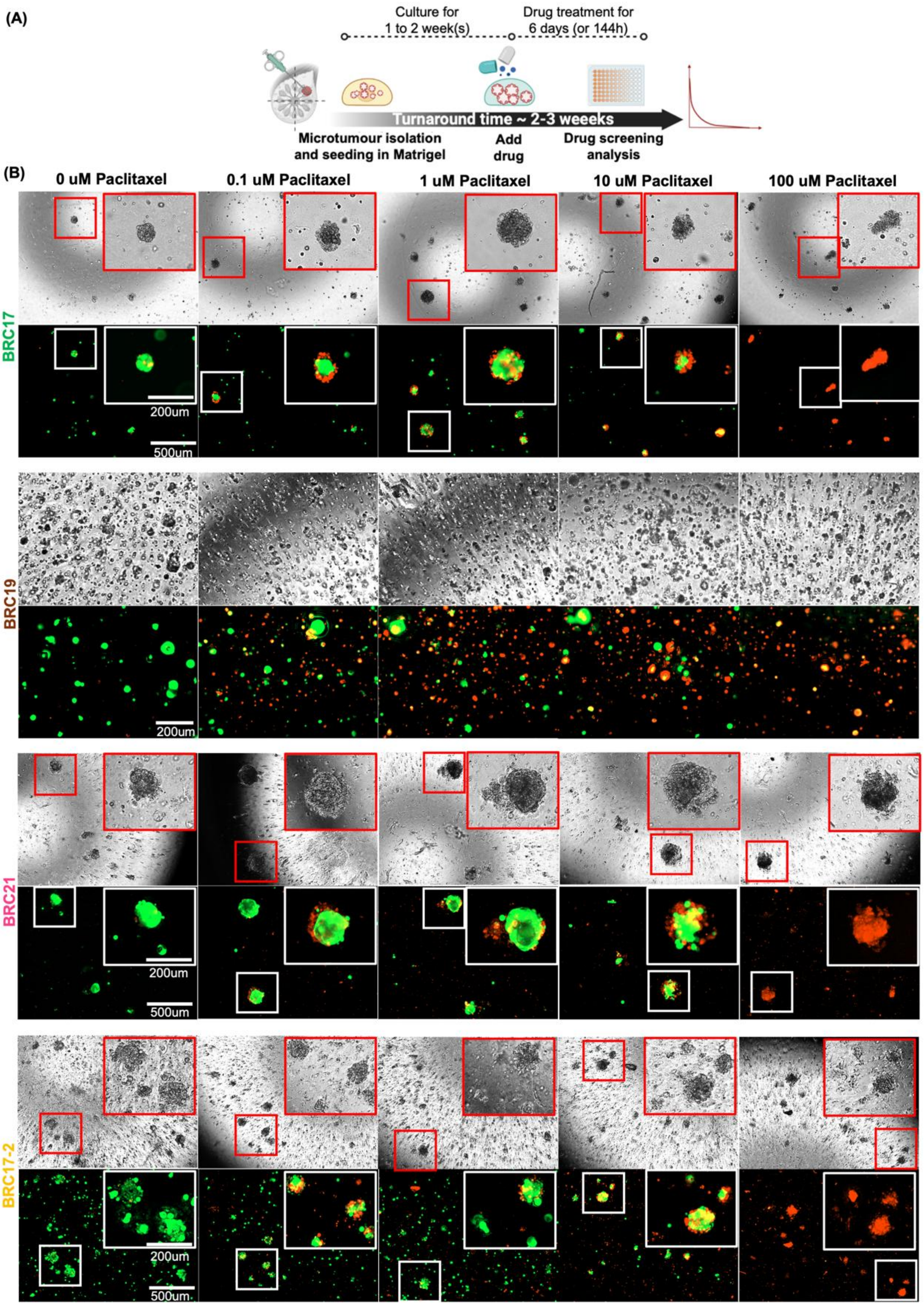

Figure S2. Paclitaxel treatment of breast cancer PDMs

(A) The diagram image depicted in a drug screening timeline from surgery to analysis results within 2 to 3 weeks. (B) The fluorescent images of Calcein AM (live indicator in green color) and BOBO-3 iodide (dead indicator in red color) staining presented the microtumour sensitivity to paclitaxel after 144h treatment (each condition containing at least 5 active microtumours per well, enabling tracking growth rates along the growing period, from total 4 analyzed cases).

Table S1. The characteristics of tissue specimens

Cases	Ages	Sample types	Clinical status	Pre-treatments	TNM	Stages	Histological analysis	Grades	ER	PR	HER2
BRC16	49	Biopsy	New case	No	T4bN0M1	4	IDC	2	100%	100%	0
BRC17	71	Biopsy	Relapsed case	BCS+FAC in 2012	T3N0M0	2B	IDC with mucinous	3	100%	0	0
BRC18	52	Biopsy	New case	No	T4bN1M1	4	IDC	2	100%	100%	0
BRC19	84	MRM	New case	No	T4bN1M0	3B	IDC	2	0	0	0
BRC20	56	Biopsy	New case	No	T4bN1M0	3B	IDC	3	0	0	3+
BRC21	48	Biopsy	New case	No	T4bN1M0	3B	IDC	2	2% (low positive)	0	3+
BRC22	54	Biopsy	New case	No	T2N1M0	2B	IDC	3	100%	50%	3+
BRC17-2	72	Mastectomy	Relapsed case	BCS+FAC in 2012 LET in current	T3N0M0	2B	IDC with mucinous	3	100%	0	0

Note: BCS= Breast conserving surgery, FAC= Combined 5-fluorouracil, doxorubicin and cyclophosphamide, LET= Letrozole, TNM= Tumour/ Lymph Nodes/ Metastasis, IDC= Invasive Ductal Carcinoma, ER= Estrogen receptor, PR= Progesterone receptor, HER2= Human epidermal growth factor receptor 2, MRM= Modified radical mastectomy

Table S2. The clinical treatment of patient enrollments

Patient cases	Neoadjuvant drug treatments	Adjuvant drug treatments	Palliative treatments	Patient responses	Surgery after drug responses
BRC16	-	-	LET+Ribociclib	Complete response	-
BRC17 and BRC17-2	LET	-	-	Partial response	Mastectomy
BRC18	-	-	Tamoxifen	Loss follow-up	-
BRC19	- (Cannot treatment with chemotherapy as 59% LVEF)	-	-	-	MRM
BRC20	AC4+PTH4	-	-	Complete response	BCS
BRC21	TdxD4+PTH4	-	-	Complete response	MRM
BRC22	TdxD4+PTH4	-	-	Complete response	MRM

Note: LET= Letrozole (hormone therapy), AC= Cyclophosphamide and Doxorubicin, PTH= Pertuzumab (P), Paclitaxel (T), and Trastuzumab (H), TdxD=Trastuzumab deruxtecan, MRM= Modified radical mastectomy, BCS= Breast-conserving surgery, LVEF= Abnormal left ventricular ejection fraction
